# A Novel IMU Extrinsic Calibration Method for Mass Production Land Vehicles

**DOI:** 10.3390/s21010007

**Published:** 2020-12-22

**Authors:** Vicent Rodrigo Marco, Jens Kalkkuhl, Jörg Raisch, Thomas Seel

**Affiliations:** 1 Control Systems Group, Department of Electrical Engineering and Computer Science, Technische Universität Berlin, D-10587 Berlin, Germany; raisch@control.tu-berlin.de (J.R.); thomas.seel@tu-berlin.de (T.S.); 2 Research and Development, Daimler AG, 71059 Sindelfingen, Germany; jens.c.kalkkuhl@daimler.com

**Keywords:** extrinsic calibration, motion estimation, automotive industry, nonlinear systems, inertial sensors, Kalman filter, odometry, autonomous driving, simultaneous state and parameter estimation, systems and control engineering

## Abstract

Multi-modal sensor fusion has become ubiquitous in the field of vehicle motion estimation. Achieving a consistent sensor fusion in such a set-up demands the precise knowledge of the misalignments between the coordinate systems in which the different information sources are expressed. In ego-motion estimation, even sub-degree misalignment errors lead to serious performance degradation. The present work addresses the extrinsic calibration of a land vehicle equipped with standard production car sensors and an automotive-grade inertial measurement unit (IMU). Specifically, the article presents a method for the estimation of the misalignment between the IMU and vehicle coordinate systems, while considering the IMU biases. The estimation problem is treated as a joint state and parameter estimation problem, and solved using an adaptive estimator that relies on the IMU measurements, a dynamic single-track model as well as the suspension and odometry systems. Additionally, we show that the validity of the misalignment estimates can be assessed by identifying the misalignment between a high-precision INS/GNSS and the IMU and vehicle coordinate systems. The effectiveness of the proposed calibration procedure is demonstrated using real sensor data. The results show that estimation accuracies below 0.1 degrees can be achieved in spite of moderate variations in the manoeuvre execution.

## 1. Introduction

Accurate and reliable information of the vehicle ego-motion is pivotal for the proper operation of active safety and automated driving functionalities. Hence, nowadays vehicles are equipped with sensors devoted to the estimation of the vehicle motion. In order to meet the high accuracy and reliability demands, multi-modal sensor fusion has become ubiquitous in the field of vehicle motion estimation [1,2,3]. Sensors relying on different technologies, with complementary benefits and deficiencies, are combined in order to increase the overall accuracy and reliability of the motion estimation solution.

In a multi-modal sensor fusion set-up, the individual information sources supply motion quantities that generally relate to different points within the vehicle and are expressed in different coordinate systems. A consistent fusion of the measurements demands that the supplied motion variables relate to and are represented in a common coordinate system. Hence, the transformations between the individual coordinate systems need to be known. In automotive series projects, the position and orientation of the sensors is usually precisely defined in a computer aided design (CAD) of the vehicle. Nevertheless, tolerances in manufacturing technologies may cause the location and orientation of the sensors to significantly differ for the real vehicles. For instance, from our experience, in car mass production, IMU mounting errors of few millimetres and two to three degrees can be expected. These inaccuracies in the transformations between the individual coordinate systems deteriorate the performance of fusion algorithms and may even cause the estimator to diverge [4]. Therefore, many applications require calibration procedures that estimate the transformations between the different coordinate systems. This process is also known in the literature by extrinsic calibration [5,6].

In the last few decades, the topic of extrinsic calibration has received increasing attention due to its significance in relevant engineering fields such as robotics. A considerable amount of literature covering the extrinsic calibration of systems involving perception sensors has been produced, e.g., [7,8,9,10,11,12]. Comparatively, the extrinsic calibration of systems not reliant on perception sensors has received considerably less attention. Motion estimation schemes fusing information from odometers (based on wheel speed and steering angle sensors), vehicle models and inertial sensors have been broadly used in active safety and automated driving technologies [3,13,14]. In such a set-up, the IMU angular rates and specific forces are given in the coordinate system of the IMU while the information supplied by the combination of odometers with kinematic [15] and dynamic models [16] is customarily expressed in a vehicle coordinate system. The identification of the relative pose between the IMU and the vehicle coordinate system is rarely addressed in the available vehicle motion estimation algorithms. Instead, a perfect alignment or known extrinsic calibration parameters are commonly assumed [15,17,18]. While, considering the low angular motion of land vehicles, the positioning errors may be neglected, the IMU misalignment errors have a significant impact on the motion estimates [19].

### 1.1. Brief Review of Previous Approaches

Although scarce, some literature has been produced to address the estimation of the IMU–vehicle extrinsic calibration. Most of the proposed approaches rely on the non-holonomic constraint [20,21,22,23,24], which assumes zero lateral and vertical velocity in the rear axle. However, differences are found with regard to the sensor portfolio and the quality of the sensors used, as well as the parameters identified.

The methods presented in [20] address the estimation of the mounting misalignments of an IMU (roll, pitch and yaw) using the accelerometer signals together with GPS-derived velocities and the non-holonomic constraint. First, the pitch and roll misalignment angles are estimated. Then, these estimates are used for the estimation of the yaw misalignment. Accelerometer biases, tilted roads and accelerations during the roll/pitch calibration phase will result in errors in the estimated misalignment angles. Estimation errors of up to 2 degrees are reported, which are prohibitively large for applications requiring highly accurate motion state estimates.

Some researchers have proposed methods to identify the misalignment angles between the IMU and vehicle coordinate system using IMU, odometry, GNSS information and the non-holonomic constraint [21,22]. For instance, [22] describes an approach that estimates the IMU pitch and yaw misalignment angles using a GNSS/INS aided dead reckoning approach. The method is experimentally tested with data obtained from typical navigation-grade, tactical-grade and low-cost (automotive-grade) IMUs. For the navigation- and tactical-grade IMUs, the misalignment estimates rapidly converge. However, the estimates for the low-cost IMU display a less steady behaviour. Despite its widespread use, GNSS is susceptible to interference and it lacks reliability in some environments. Moreover, not all land vehicles are equipped with an accurate GNSS receiver.

Aware of these drawbacks, some research groups have developed self-contained methods that perform the IMU–vehicle extrinsic calibration, solely reliant on odometry and IMU signals [23,24,25]. In [23], Xue et al. propose an in-motion alignment algorithm that estimates the vehicle-IMU misalignment (pitch and yaw) together with a unique scaling factor for the entire odometry. The method relies on the non-holonomic constraint, odometry measurements and a high-precision 6D IMU that allows north alignment via gyrocompassing. Furthermore, it assumes perfect alignment between the road plane and the vehicle body. A similar approach is proposed in [24], which, based on the same information sources, additionally estimates the IMU position with respect to the vehicle’s rear axle. Despite the accuracy shown in the experimental results of [23,24], tactical- or navigation-grade IMUs are prohibitively costly for car series projects, limiting their use for research and development purposes.

The previously presented approaches rely on the non-holonomic constraint. The main drawbacks of such methods is that when the vehicle does not comply with the non-holonomic constraint, e.g., when a side-slip angle builds during cornering or a vertical movement is caused by the suspension, errors are transferred to the misalignments estimates [22]. Furthermore, in most cases, the roll misalignment is neglected since, due to the non-holonomic constraint, it is unobservable. One of the few works that is not based on the non-holonomic constraint and relies on an automotive-grade IMU is [25]. The approach proposed in this work estimates the longitudinal and lateral accelerometer biases as well as the IMU–vehicle yaw misalignment using FIR-Filter modulating functions and an algebraic observer. Instead of applying the non-holonomic constraints, vertical and lateral velocity measurements are used. These measurements are built upon suspension signals and a single-track model. Nevertheless, the supplied estimates exhibit a relatively unsteady behaviour, which raises questions regarding noise robustness and parameter identifiability.

All in all, there is a scarcity of methods which, not relying on accurate GNSS information and based on an automotive-grade IMU, provide accurate estimates of all three misalignment angles (roll, pitch and yaw). Another shortcoming of the state-of-the-art is that most of the previous approaches assume a perfect alignment between vehicle body and road surface. Changes in the suspension state, due to effects such as variations in the load distribution or speed, are taken as variations in the IMU pitch misalignment, e.g., [22,24]. This demands a continuous estimation of the misalignment angles. Therefore, a method that takes into account suspension information would be desirable. By doing so, an adjustment of the IMU misalignment estimation would only be required after structural changes in the platform, such as a replacement of the IMU or modifications in the steering system.

Besides the already discussed challenges, one of the aspects to consider is the fact that the real IMU misalignment is usually not known. Hence, there is a lack of a ground-truth reference against which the estimates can be directly compared. [22] proposes to validate the estimation by checking that the estimated velocity (expressed in the vehicle coordinate system) has no significant lateral and vertical velocity components when the misalignments have been compensated. This validation is consistent as long as, in the analysed data, the non-holonomic constraint is satisfied, which will not be the case when turning or driving on a road with significant bank angles. Another option is presented in [24], in which the correctness of the estimated calibration parameters is assessed by analysing the improvement of the position error of the dead-reckoning solution when the calibration parameters have been corrected. However, this approach does not provide a direct way to assess the errors of the misalignment estimates.

### 1.2. Current Approach and Main Contributions

This manuscript proposes an estimation approach that addresses the identification of the 3D rotation between the IMU and vehicle coordinate systems, which is fully determined by three misalignment angles (roll, pitch and yaw). In order to avoid estimation inaccuracies stemming from the IMU biases, these are compensated in the algorithm. The approach is aimed at automotive series projects and, hence, the use of prohibitively expensive sensors is avoided. Therefore, the sensor portfolio is comprised by an automotive-grade 6D IMU and series chassis sensors.

Additionally, for analysis and evaluation purposes, test vehicles are commonly equipped with high accuracy measurement systems, which supply ground-truth motion signals. In our test vehicle, a high-precision INS/GNSS deeply coupled inertial navigation solution is mounted. This article also presents a method to estimate the 3D rotation between the ground-truth and both the IMU and vehicle coordinate systems. These estimates are not only necessary to assess the performance of motion estimation algorithms but also serve as a validation of the 3D rotation estimate between the IMU and the vehicle coordinate systems. Please note that the misalignment between the IMU and the vehicle coordinate systems may be obtained from the 3D rotation between the ground-truth and IMU coordinate systems, and the 3D rotation between the ground-truth and the vehicle coordinate systems.

Based upon the foregoing, the main contributions of this article are listed below.

- The proposed estimation scheme estimates all three misalignment angles while considering the IMU biases. This contrasts with most previously proposed approaches, in which the roll misalignment is neglected.
- A calibration manoeuvre is proposed for which a persistent excitation condition for the identification of the misalignment angles is experimentally validated. Furthermore, it is shown that the estimation results remain consistent despite moderate variations in the manoeuvre execution.
- The estimation scheme relies on cost-efficient automotive sensors, and it is not dependent on neither perception nor GNSS information. Experimental results show that, in spite of not using high-precision measurement systems, the approach supplies accurate and reliable misalignment estimates.
- Instead of relying on the non-holonomic constraint, information from the suspension system and a single-track model is used. Hence, the approach considers vertical movements caused by the suspension and side-slip angles in the rear axle, e.g., built during cornering or while driving on a road with significant bank angles.
- The pose of the vehicle with respect to the road plane is not seen as an IMU misalignment. Hence, readjustment of the misalignment estimates is not required due to, for instance, load redistribution or changes in the speed. This reduces the need for a continuous extrinsic calibration. A re-estimation of the misalignment angles would just be required after structural modifications in the platform, e.g., replacement of the IMU or alterations in the odometry.
- An approach to directly assess the validity of the misalignment estimates is proposed. This is based on the identification of the 3D rotation between a high-precision INS/GNSS, taken as ground-truth, and the IMU and vehicle coordinate systems.

**Figure 1:**
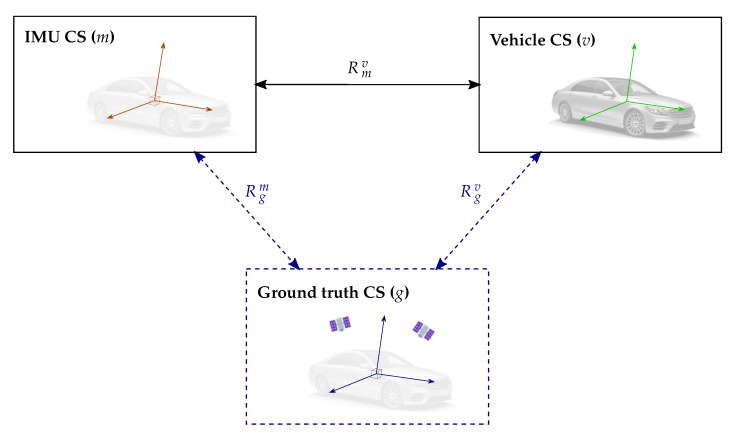
The present work proposes an approach to estimate the 3D rotation between the IMU and vehicle coordinate systems ($R_{\;m}^{\;v}$). Additionally, calibration procedures are described to determine the 3D rotation between a ground-truth measurement system and both the IMU and vehicle coordinate systems ($R_{\;g}^{\;m}$ and $R_{\;g}^{\;v}$, respectively). These represent a reference to directly assess the accuracy of the estimated IMU–vehicle 3D rotation.

In short, the current work presents an extrinsic calibration procedure that supplies the 3D rotations between the ground-truth, IMU and vehicle coordinate systems (see Figure 1).

The paper is organised as follows. In Section 2, the required coordinate system definitions, the used notation as well as some preliminaries are introduced. The ground-truth–vehicle, ground-truth–IMU and IMU–vehicle extrinsic calibration procedures are presented in Section 3, Section 4 and Section 5, respectively. Each one of the proposed estimation schemes is experimentally validated in its corresponding section. Finally, a summary and some important remarks conclude this article (Section 6).

## 2. Coordinate Systems and Preliminaries

### 2.1. Coordinate Systems

**Figure 2:**
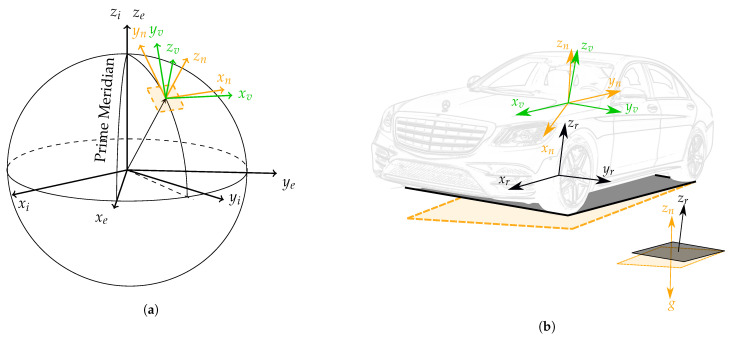
Representation of the inertial (*i*), ECEF (*e*), local navigation (*n*), vehicle (*v*) and road (*r*) coordinate systems. For a clear depiction, a world and vehicle perspectives are displayed, which corresponds with (**a**,**b**), respectively. The black and yellow planes respectively represent the road plane and the plane tangent to the Earth’s ellipsoid, which is considered to be normal to gravity. Please note that *n* and *v* share the same origin, and the origin of *r* lies on the road plane.

**Figure 3:**
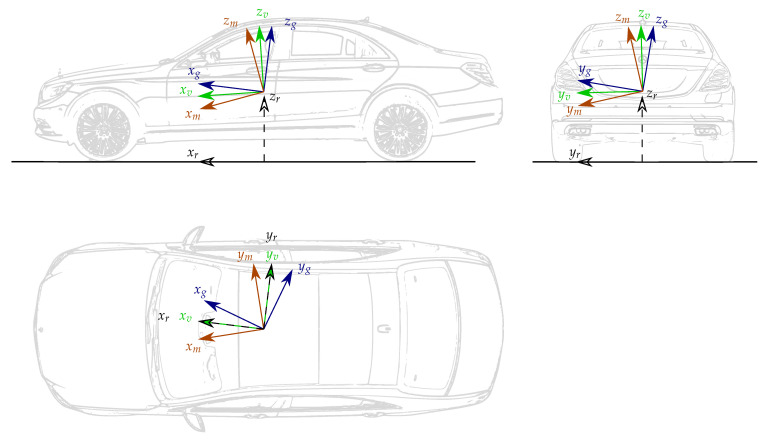
Representation of the ground-truth (*g*), IMU (*m*), vehicle (*v*) and road (*r*) coordinate systems. Please note that the origin of the ground-truth and IMU coordinate systems is not necessarily coincident with that of the vehicle coordinate system. This representation has been chosen to highlight the misalignment between the different coordinate systems.

In order to describe the position, orientation and motion of a vehicle based on the information supplied by the available sensors and vehicle models, some coordinate systems (CS) need to be introduced, see Figure 2 and Figure 3. Besides coordinate systems commonly used in the navigation literature [26], i.e., the *inertial coordinate system* (*i*), the *Earth-Centred Earth-Fixed coordinate system* (*e*) as well as the *local navigation coordinate system* (East-North-Up) (*n*), some additional coordinate systems are defined.

The *vehicle coordinate system* (*v*) is fixed to the vehicle sprung mass and its origin is located on the longitudinal symmetry plane of the vehicle at the mid-wheelbase point at the height of the center of mass (in reference loading conditions). Regarding the orientation of the axes, the $x_{v}$- and $y_{v}$-axes lie on a plane defined in the CAD design of the vehicle, and the $z_{v}$-axis is normal to this plane pointing upwards. The $y_{v}$-axis points to the left, and the $x_{v}$-axis points forward. Nevertheless, unlike [27], the $x_{v}$-axis does not necessarily lie on the longitudinal symmetry plane of the vehicle. This specific orientation is determined by the used dynamic model. In the present work, it is aligned with the direction of travel while driving on a horizontal road with no vertical angular rate, conditions under which the single-track model supplies zero lateral velocity. Note that, due to asymmetries in the vehicle, this direction of travel does not necessarily lie on the theoretical symmetry plane of the vehicle.

The *IMU* and *ground-truth coordinate systems* (*m* and *g*, respectively) are fixed to the sprung mass of the vehicle. Their origins and axes orientations are determined by the mounting position and orientation of the IMU and ground-truth systems, respectively. In the test vehicle used in this work, the *x*-, *y*-, and *z*-axes point in the forward direction of the vehicle, to the left and upward, respectively. Due to mounting errors, their axes are not aligned.

The origin of the *road coordinate system* (*r*) is located on the *road plane* (The *road plane* is a plane that represents the road surface, on which the tyres are supported and which produces the friction required to move the vehicle. In case of planar road surfaces, the road plane is coincident with the road surface. Nevertheless, if the road surface contours have a wavelength similar to or less than the size of the vehicle, an equivalent road plane is determined, which is an approximation of the actual road surface. The road plane is determined by a best fit approximation through the tire contact patches.) maintaining the symmetry with respect to the vehicle wheels. The $z_{r}$-axis of the road coordinate system is normal to the road plane and its $x_{r}$-axis points in the direction of the $x_{v}$-axis.

### 2.2. Notation

The present paper adopts the notation commonly used in the navigation systems literature [26,28]. Specifically, three indices are employed to characterize motion variables, such as velocities v or angular rates ω. Let us illustrate this with the following angular rate:

(1) $$\omega_{\;in}^{\;v}=\left(\begin{array}{c} \omega_{\;in,x}^{\;v}\\ \omega_{\;in,y}^{\;v}\\ \omega_{\;in,z}^{\;v} \end{array}\right)$$

The superscript index specifies the coordinate system in which the motion quantity is decomposed (*resolving coordinate system*). The right lower index indicates the coordinate system whose motion is described (*object coordinate system*). Finally, the left lower index denotes the coordinate system with which the motion is respect to (*resolving coordinate system*). Hence, $\omega_{\;in}^{\;v}$ depicts the angular rate of the local navigation coordinate system with respect to the inertial coordinate system resolved in the vehicle coordinate system.

The relative attitude between two coordinate systems may be represented via Euler angles. Particularly, intrinsic Euler angles with the ZYX convention are adopted in the present work. Attitude is fully described by the object and reference coordinate systems and, therefore, Euler angles are typically denoted using two indices. For example:

(2) $$\psi_{nv}=\left(\begin{array}{c} \phi_{\;nv}\\ \theta_{\;nv}\\ \psi_{\;nv} \end{array}\right)$$

represents the Euler rotation from the local navigation coordinate system to the vehicle coordinate system. Here, the first rotation, $\psi_{nv}$, called yaw, is performed about $z^{\;n}$-axis; the second rotation, $\theta_{nv}$, known as pitch, is performed about the once rotated *y*-axis; the third rotation, $\phi_{nv}$, also named roll, is performed about the twice rotated *x*-axis. The corresponding rotation matrix $R_{\;n}^{\;v}$ transforms a motion quantity from the set of resolving axes of the coordinate system *n* to those of the coordinate system *v*:

(3) $$\omega_{\;iv}^{\;v}=R_{\;n}^{\;v}\;\omega_{\;iv}^{\;n}$$

For small rotations, for which the small-angle approximation holds (sinα≈0, cosα≈1, sinαsinα≈αα≈0), some approximations can be made:

(4) $$R_{\;n}^{\;v}\approx \left(\begin{array}{ccc} 1 & \psi_{nv} & -\theta_{nv}\\ -\psi_{nv} & 1 & \phi_{nv}\\ \theta_{nv} & -\phi_{nv} & 1 \end{array}\right)\;\;\;\;\;\;\;\;\;\mathrm{where}\;\;\;\;\;\;\;\;\;\left(\begin{array}{c} \phi_{nv}\\ \theta_{nv}\\ \psi_{nv} \end{array}\right)\approx \left(\begin{array}{c} -\phi_{vn}\\ -\theta_{vn}\\ -\psi_{vn} \end{array}\right)$$

### 2.3. Inertial Measurement Unit: Preliminaries

A 6D IMU is a sensor supplying measurements of the angular rate and specific force of the vehicle body (at the mounting location) with respect to the inertial coordinate system, i.e., $\omega_{\;im}^{\;m}$ and $f_{\;im}^{\;m}$. While the angular rate is the same within a rigid body (e.g., $\omega_{\;im}^{\;m}=\omega_{\;iv}^{\;m}=\omega_{\;ig}^{\;m}$, hereafter $\omega^{\;m}$), the specific force of a rotating body depends on the particular point within the body (e.g., $f_{\;im}^{\;m}\neq f_{\;iv}^{\;m}\neq f_{\;ig}^{\;m}$). When the relative location between a point of interest and the mounting position of an IMU is known, the specific force may be transformed to the point of interest using a simple kinematic equation [17]. Additionally, the signals supplied by the IMU are corrupted by different error sources such as biases, scaling factors and non-orthogonalities [29]. This work assumes that most errors have been corrected at an end-of-line calibration of the IMU [30]. However, for the automotive-grade IMU, biases are further considered due to the significant turn-on and in-run terms [26]. Hence, the following IMU error model is considered:

(5) $$\begin{array}{cc} {\tilde{f}}_{\;im}^{\;m} & =f_{\;im}^{\;m}+b_{f} \end{array}$$

(6) $$\begin{array}{cc} {\tilde{\omega }}^{\;m} & =\omega^{\;m}+b_{\omega }, \end{array}$$

where ${\tilde{f}}_{\;im}^{\;m}$ and ${\tilde{\omega }}^{\;m}$ denote the measured specific force and angular rates, and  $b_{f}$ and $b_{\omega }$ the accelerometer and gyro biases, respectively.

## 3. Ground-Truth–Vehicle Misalignment Estimation

As previously stated, the precise knowledge of the ground-truth–vehicle relative pose is not only essential for analysis and evaluation purposes but, together with the ground-truth–IMU pose, provides a reference for the IMU–vehicle misalignment estimation. In this section, a method to estimate the misalignment angles between the ground-truth and vehicle coordinate systems ($\phi_{vg}$, $\theta_{vg}$, $\psi_{vg}$) is proposed and experimentally validated. The method can be divided into two parts, i.e., the estimation of the pitch and roll misalignments, and the estimation of the yaw misalignment.

The employed ground-truth is a high-precision INS/GNSS deeply coupled inertial navigation system, which provides accurate velocities, orientation angles ($\phi_{ng}$, $\theta_{ng}$, $\psi_{ng}$), specific forces and angular rates.

### 3.1. Roll and Pitch Misalignment

The roll and pitch misalignment angles ($\phi_{vg}$, $\theta_{vg}$) may be determined while standing still, thanks to the high-precision IMU installed in the ground-truth system, as well as the vehicle level measurements.

**Figure 4:**
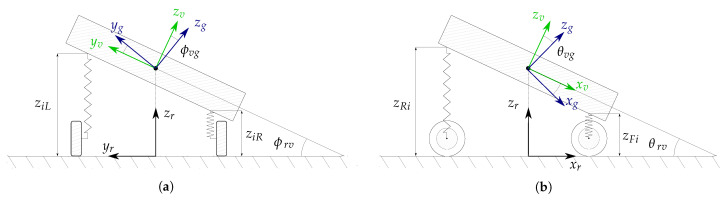
Vehicle-to-road pose [3]. (**a**) shows the roll model, where i∈[F,R]; (**b**) depicts the pitch model where i∈[L,R].

The spring deflections, supplied by the vehicle level sensors, together with the tyre deflections, provided by a simple tyre vertical model, are used to compute the vehicle levels over-road $z_{i}$ [3]. Here, i∈[FL,FR,RL,RR], where FL, FR, RL, and RR denotes to the front left, front right, rear left and rear right wheels, respectively. Therewith, the pitch and roll angles with respect to the road plane are obtained (see Figure 4). In most vehicles, these angles remain very small during standstill phases. Hence, the small-angle approximation is valid, sinα≈α and cosα≈1:

(7) $$\begin{array}{cc} \theta_{\;rv} & =\frac{z_{\;\mathrm{RL}}+z_{\;\mathrm{RR}}-z_{\;\mathrm{FL}}-z_{\;\mathrm{FR}}}{2\;l}\\ \phi_{\;rv} & =\frac{1}{2}\;\left(\frac{z_{\;\mathrm{FL}}-z_{\;\mathrm{FR}}}{t_{f}}+\frac{z_{\;\mathrm{RL}}-z_{\;\mathrm{RR}}}{t_{r}}\right) \end{array}$$

where *l*, $t_{F}$ and $t_{R}$ are respectively the wheel base and the front and rear track-widths.

Assuming that the vehicle is standing on a road plane with small bank and slope angles ($\phi_{nr}$, $\theta_{nr}$), and bearing in mind that the misalignment angles and the vehicle angles with respect to the road plane are small, the following relationship holds:

(8) $$\begin{array}{cc} \phi_{ng} & =\phi_{nr}+\phi_{rv}+\phi_{vg}\\ \theta_{ng} & =\theta_{nr}+\theta_{rv}+\theta_{vg}, \end{array}$$

where $\phi_{ng}$ and $\theta_{ng}$ are obtained from the ground-truth, and $\phi_{rv}$ and $\theta_{rv}$ from the vehicle levels according to (7). If the vehicle stands on a horizontal road ($\theta_{nr},\phi_{nr}=0$), the misalignment angles may be directly obtained using the following relationships:

(9) $$\begin{array}{cc} \phi_{vg} & =\phi_{ng}-\phi_{rv}\\ \theta_{vg} & =\theta_{ng}-\theta_{rv} \end{array}$$

**Figure 5:**
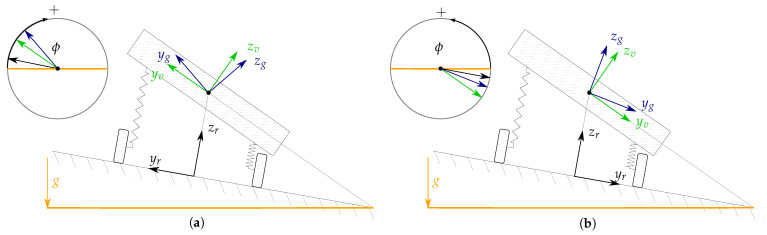
Calibration procedure on non-horizontal road (roll angle perspective). (**a**) depicts the first standstill pose and (**b**) the second, which is equal to the first pose but facing the opposite direction. *g*, *v* and *r* denote the ground-truth, vehicle and road coordinate systems, respectively. (**a**,**b**) correspond with (10) and (11), respectively. The yellow line represents the horizontal plane. The circle on the upper left shows the direction of positive roll angles, and the arrows in it represent the *y*-axes of the corresponding coordinate systems. Note that, in the circles, the angle between the yellow line and the black arrow correspond with $\phi_{nr}$. It can then be seen that $\phi_{nr,2}=-\phi_{nr,1}$. The corresponding pitch angle perspective would be equivalent. It is omitted for the sake of conciseness.

However, horizontal road surfaces are not always available. In such case, the unknown slope and bank angle can be cancelled out by using two standstill phases at the exact same position but with the vehicle facing opposite directions (see Figure 5, where Figure 5a corresponds with pose 1 and Figure 5b with pose 2):   

(10) $$\begin{array}{cc} \phi_{ng,1} & =\phi_{nr,1}+\phi_{rv,1}+\phi_{vg} \end{array}$$

(11) $$\begin{array}{cc} \phi_{ng,2} & =\phi_{nr,2}+\phi_{rv,2}+\phi_{vg}=-\phi_{nr,1}+\phi_{rv,2}+\phi_{vg} \end{array}$$

(12) $$\begin{array}{cc} \theta_{ng,1} & =\theta_{nr,1}+\theta_{rv,1}+\theta_{vg} \end{array}$$

(13) $$\begin{array}{cc} \theta_{ng,2} & =\theta_{nr,2}+\theta_{rv,2}+\theta_{vg}=-\theta_{nr,1}+\theta_{rv,2}+\theta_{vg} \end{array}$$

Adding (10) and (11), as well as (12) and (13), and reorganising the terms yields

(14) $$\begin{array}{cc} \phi_{vg} & =\frac{\phi_{ng,1}+\phi_{ng,2}-\phi_{rv,1}-\phi_{rv,2}}{2} \end{array}$$

(15) $$\begin{array}{cc} \theta_{vg} & =\frac{\theta_{ng,1}+\theta_{ng,2}-\theta_{rv,1}-\theta_{rv,2}}{2} \end{array}$$

### 3.2. Yaw Misalignment

Unlike for the pitch and roll cases, vehicle motion is required to determine the yaw misalignment between the ground-truth and the vehicle coordinate systems ($\psi_{vg}$). Note that the used single-track model partially defines the vehicle coordinate system (see Section 2):

(16) $$\begin{array}{cc} v_{\;er,y}^{r} & \approx l_{r}\;\omega_{z}^{r}-SG\;v_{\;er,x}^{r}\;f_{\;ir,y}^{r}, \end{array}$$

where $v_{\;er,x}^{r}$, $\omega_{z}^{r}$ and $f_{\;ir,y}^{r}$ represent the over-road lateral velocity, vertical angular rate and lateral specific force, respectively, and SG and $l_{r}$ are the sideslip angle gradient and the distance between the center of gravity and the rear axle. Using the roll angle with respect to the road plane, the velocity of the vehicle coordinate system resolved in vehicle coordinates may be expressed as follows:

(17) $$v_{\;ev,y}^{\;v}=v_{\;er,y}^{\;r}+v_{\;rv,z}^{\;r}\;\phi_{rv}-h_{r}\;{\dot{\phi }}_{rv}$$

**Figure 6:**
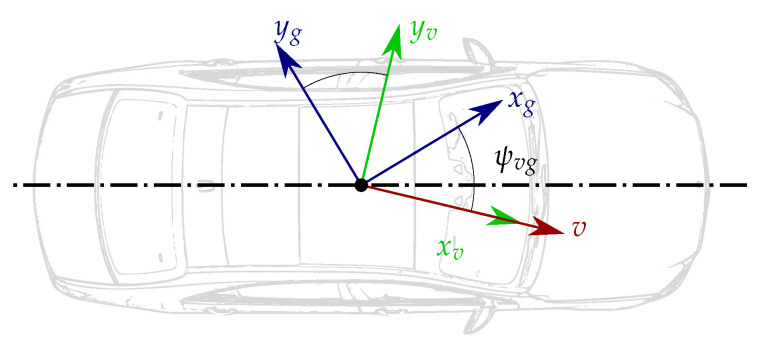
Velocities of a vehicle while driving straight ahead with constant speed over a well-paved horizontal road.

When driving straight ahead with constant speed over a well-paved horizontal road, we have $\omega_{z}^{\;r},\;v_{\;rv,z}^{\;r},\;{\dot{\phi }}_{rv},\;f_{ir,y}^{\;r}\approx 0$, and, hence, $v_{\;ev,y}^{\;v}\approx 0$. Therefore, under these conditions, the projection of the velocity vector onto the $z_{v}$-plane (plane normal to the $z_{v}$ vector) is completely aligned with $x_{v}$ (see Figure 6). Please note that, due to asymmetries in the vehicle, the $x_{v}$-axis does not necessarily lie on the longitudinal symmetry plane. In such situation, $\psi_{vg}$ can be determined by the direction in which the ground-truth perceives the velocity vector:

(18) $$\psi_{vg}=-\beta_{g},$$

where $\beta_{g}≜\arctan\frac{v_{\;ev,y}^{g}}{v_{\;ev,x}^{g}}$ can directly be obtained from the ground-truth velocity measurements.

Unfortunately, not all test tracks possess a perfectly horizontal road. Most seemingly horizontal roads have some kind of bank angle for water drainage purposes. Due to gravity, even small road bank angles cause $f_{\;ir,y}^{r}\neq 0$, which in turn, through the term $-SG\;f_{\;ir,y}^{r}\;v_{\;er,x}^{\;r}$ causes $v_{\;ev,y}^{\;v}\neq 0$. If neglected, the lateral velocity caused by the bank angle is taken as a yaw misalignment.

**Figure 7:**
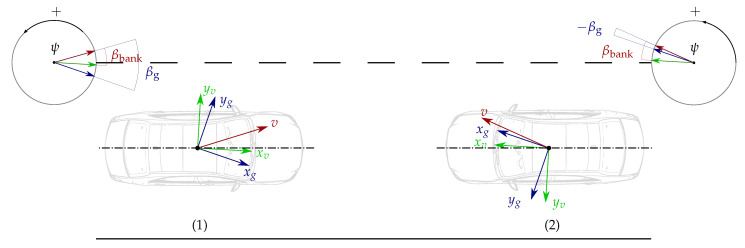
Yaw calibration procedure on a non-horizontal road. The dashed and continuous lines respectively represent the visual division between lanes and the road limit. The vehicle drives on the same lane twice but in opposite directions. The upper left and right circles show the direction of positive yaw angles for the corresponding driving directions. The red, green and blue arrows in the circle depict the directions of the velocity vector, the *x*-axis of the vehicle CS and the *x*-axis of the ground-truth CS, respectively. $\beta_{\mathrm{bank}}$ is the side-slip angle stemming from the road bank and $\beta_{g}$ the direction in which the ground-truth perceives the velocity vector.

In order to compensate the sideslip angle ($\beta_{v}≜\arctan\frac{v_{\;ev,y}^{\;v}}{v_{\;ev,x}^{\;v}}$) caused by the road bank, one can drive on the same lane twice, but in opposite directions (see Figure 7). In each drive, the sideslip angle caused by the gravity takes opposite directions:

(19) $$\begin{array}{cc} \psi_{vg} & =-\beta_{g,1}+\beta_{\mathrm{bank}} \end{array}$$

(20) $$\begin{array}{cc} \psi_{vg} & =-\beta_{g,2}-\beta_{\mathrm{bank}}, \end{array}$$

where $\beta_{\mathrm{bank}}\approx f_{ir,y}^{\;r}\;SG$, and 1 and 2 refer to the first and second drive, respectively. Hence, adding (19) and (20) yields   

(21) $$\begin{array}{cc} \psi_{vg} & =-\frac{\beta_{g,1}+\beta_{g,2}}{2}, \end{array}$$

where $\beta_{g,1}$ and $\beta_{g,2}$ are obtained from the ground-truth measurements.

### 3.3. Results

The calibration to determine the relative pose between the ground-truth and vehicle coordinate systems was performed on a test-track three times (on different dates). The road bank and slope angles were below 2 degrees. For the pitch and roll angle misalignment procedure, the vehicle was kept at a standstill for at least 30s in each direction. In order to avoid movement in the car, the driver and passengers got out of the car in both standstill phases. The values $\theta_{ng,1}$, $\theta_{ng,2}$, $\theta_{rv,1}$ and $\theta_{rv,2}$ were obtained by averaging over the standstill phase.

For the yaw misalignment manoeuvre, the vehicle was accelerated to 30 km/h and kept at constant speed for at least 100 m. Afterwards, the vehicle was turned 180 deg and driven back along the same lane at the same velocity (see Figure 7). The values $\beta_{g,1}$ and $\beta_{g,2}$ were obtained by averaging over the samples corresponding to the constant speed phase. The results are depicted in Table 1:

The maximum difference among the misalignment estimates within the three calibration procedures lies below 0.03 deg for both the roll ${\hat{\phi }}_{vg}$ and pitch ${\hat{\theta }}_{vg}$ misalignment angles. A bit larger is the variability of the yaw misalignment estimate ${\hat{\psi }}_{vg}$, presenting a maximum difference of 0.055 deg. In any case, the variability remains low and, hence, it can be concluded that the estimation results are consistent.

## 4. Ground-Truth–IMU Misalignment Estimation

One of the main challenges faced when addressing the estimation of the misalignment between an automotive-grade and a ground-truth IMU is the fact that the biases of the automotive-grade IMU must be considered. Hence, the complexity of the problem rises to that of estimating both the biases and misalignment angles. In the present section, an approach is presented that addresses the misalignment estimation between the IMU (*m*) and the ground-truth (*g*) coordinate systems while considering the biases of the automotive-grade IMU.

### 4.1. Method

**Figure 8:**
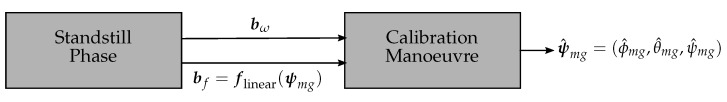
Calibration procedure for the estimation of the misalignment between the ground-truth and IMU coordinate systems.

The proposed calibration procedure consists of two successive phases, in which the outputs of both the automotive-grade and ground-truth IMUs are used. First, during a standstill phase, the angular rate biases are determined and linear constraints between the accelerometer biases and the misalignment angles are identified. Then, a calibration manoeuvre is performed, which provides enough excitation to estimate the misalignment angles (see Figure 8). The fact that the automotive-grade and ground-truth IMUs are physically mounted at different positions within the vehicle body is taken into account. The ground-truth specific force signals ($f_{ig}^{g}$) are transformed to the mounting position of the automotive-grade IMU ($f_{im}^{g}$) in a pre-processing step.

#### 4.1.1. Standstill Phase

During standstill, the angular rate biases can be determined relatively easily [31]. However, this is not the case for the accelerometer biases. On the one hand, the pitch and roll angles of the IMU coordinate system *m* with respect to the local navigation coordinate system *n* are not perfectly known and, on the other hand, the discrepancies between the outputs of the ground-truth and the automotive-grade IMUs stem from a combination of bias and misalignment errors. Even though the accelerometer biases cannot be directly identified during standstill, a relationship between the bias and misalignment errors can be identified.

The misalignment angles are assumed to be small. Hence, using the small angle approximation, the rotation matrix $R_{\;m}^{\;g}$ is defined by

(22) $$R_{\;m}^{\;g}=\left(\begin{array}{ccc} 1 & \psi_{mg} & -\theta_{mg}\\ -\psi_{mg} & 1 & \phi_{mg}\\ \theta_{mg} & -\phi_{mg} & 1 \end{array}\right)$$

Furthermore, using the coordinate transformation of the specific forces from the resolving coordinate system *g* to the resolving coordinate system *m* yields:

(23) $$\begin{array}{c} \left(\begin{array}{c} f_{im,x}^{m}\\ f_{im,y}^{m}\\ f_{im,z}^{m} \end{array}\right)=\left(\begin{array}{ccc} 1 & -\psi_{mg} & \theta_{mg}\\ \psi_{mg} & 1 & -\phi_{mg}\\ -\theta_{mg} & \phi_{mg} & 1 \end{array}\right)\left(\begin{array}{c} f_{im,x}^{g}\\ f_{im,y}^{g}\\ f_{im,z}^{g} \end{array}\right), \end{array}$$

where ${\left(f_{im,x}^{g},f_{im,y}^{g},f_{im,z}^{g}\right)}^{T}$ represents the specific force resolved in ground-truth coordinates, obtained from the ground-truth measurement system, and  ${\left(f_{im,x}^{m},f_{im,y}^{m},f_{im,z}^{m}\right)}^{T}$ the specific force resolved in IMU coordinates, which is not directly accessible due to the IMU accelerometer biases. Instead, the bias-corrupted specific force signals ${\left({\tilde{f}}_{im,x}^{m},{\tilde{f}}_{im,y}^{m},{\tilde{f}}_{im,z}^{m}\right)}^{T}$ are available:

(24) $$\begin{array}{cc} {\tilde{f}}_{im,x}^{m} & =f_{im,x}^{m}+b_{f_{x}}\\ {\tilde{f}}_{im,y}^{m} & =f_{im,y}^{m}+b_{f_{y}}\\ {\tilde{f}}_{im,z}^{m} & =f_{im,z}^{m}+b_{f_{z}}. \end{array}$$

Combining (24) and (23), the biases are expressed as a function of the misalignment angles as follows:

(25) $$\begin{array}{c} \left(\begin{array}{c} b_{f_{x}}\\ b_{f_{y}}\\ b_{f_{z}} \end{array}\right)=\left(\begin{array}{c} {\tilde{f}}_{im,x}^{m}\\ {\tilde{f}}_{im,y}^{m}\\ {\tilde{f}}_{im,z}^{m} \end{array}\right)-\left(\begin{array}{c} f_{im,x}^{g}\\ f_{im,y}^{g}\\ f_{im,z}^{g} \end{array}\right)+\left(\begin{array}{ccc} 0 & -f_{im,z}^{g} & f_{im,y}^{g}\\ f_{im,z}^{g} & 0 & -f_{im,x}^{g}\\ -f_{im,y}^{g} & f_{im,x}^{g} & 0 \end{array}\right)\;\left(\begin{array}{c} \phi_{mg}\\ \theta_{mg}\\ \psi_{mg} \end{array}\right). \end{array}$$

This relationship between the accelerometer biases and misalignment angles is exploited during a standstill phase in order to set the following constraints:   

(26) $$\begin{array}{c} \left(\begin{array}{c} b_{f_{x}}\\ b_{f_{y}}\\ b_{f_{z}} \end{array}\right)=\left(\begin{array}{c} {\tilde{f}}_{im,x\;0}^{m}\\ {\tilde{f}}_{im,y\;0}^{m}\\ {\tilde{f}}_{im,z\;0}^{m} \end{array}\right)-\left(\begin{array}{c} f_{im,x\;0}^{g}\\ f_{im,y\;0}^{g}\\ f_{im,z\;0}^{g} \end{array}\right)+\left(\begin{array}{ccc} 0 & -f_{im,z\;0}^{g} & f_{im,y\;0}^{g}\\ f_{im,z\;0}^{g} & 0 & -f_{im,x\;0}^{g}\\ -f_{im,y\;0}^{g} & f_{im,x\;0}^{g} & 0 \end{array}\right)\;\left(\begin{array}{c} \phi_{mg}\\ \theta_{mg}\\ \psi_{mg} \end{array}\right), \end{array}$$

where (${\tilde{f}}_{im,x\;0}^{m}$, ${\tilde{f}}_{im,y\;0}^{m}$, ${\tilde{f}}_{im,z\;0}^{m}$) and ($f_{im,x\;0}^{g}$, $f_{im,y\;0}^{g}$ and $f_{im,z\;0}^{g}$) respectively represent the mean of the IMU and ground-truth specific force measurements during the standstill phase.

Despite the time-varying nature of the biases, it can be assumed that, in the absence of sensor malfunctions and strong changes in the environment, the biases practically remain constant within intervals of several minutes. Hence, it is a sensible assumption that the biases and constraints hold during the subsequent calibration manoeuvre.

#### 4.1.2. Calibration Manoeuvre

The misalignment angles between the IMU and ground-truth coordinate systems are estimated during a calibration manoeuvre using an estimator based on the recursive least squares (RLS) algorithm. The estimator exploits both the bias-corrected angular rates from the automotive-grade IMU, as well as the constraints identified during the standstill phase.

Applying the coordinate transformation of the angular rates and specific forces from the set of resolving axes *m* to the set of resolving axes *g* leads to the following equations:

(27) $$\left(\begin{array}{c} \omega_{x}^{g}\\ \omega_{y}^{g}\\ \omega_{z}^{g} \end{array}\right)-\left(\begin{array}{c} \omega_{x}^{m}\\ \omega_{y}^{m}\\ \omega_{z}^{m} \end{array}\right)=\left(\begin{array}{ccc} 0 & -\omega_{z}^{m} & \omega_{y}^{m}\\ \omega_{z}^{m} & 0 & -\omega_{x}^{m}\\ -\omega_{y}^{m} & \omega_{x}^{m} & 0 \end{array}\right)\left(\begin{array}{c} \phi_{mg}\\ \theta_{mg}\\ \psi_{mg} \end{array}\right)$$

(28) $$\left(\begin{array}{c} f_{im,x}^{g}\\ f_{im,y}^{g}\\ f_{im,z}^{g} \end{array}\right)-\left(\begin{array}{c} f_{im,x}^{m}\\ f_{im,y}^{m}\\ f_{im,z}^{m} \end{array}\right)=\left(\begin{array}{ccc} 0 & -f_{im,z}^{m} & f_{im,y}^{m}\\ f_{im,z}^{m} & 0 & -f_{im,x}^{m}\\ -f_{im,y}^{m} & f_{im,x}^{m} & 0 \end{array}\right)\left(\begin{array}{c} \phi_{mg}\\ \theta_{mg}\\ \psi_{mg} \end{array}\right),$$

where ${\left(\omega_{x}^{g},\omega_{y}^{g},\omega_{z}^{g}\right)}^{T}$ and ${\left(\omega_{x}^{m},and\;\omega_{y}^{m},\omega_{z}^{m}\right)}^{T}$ respectively correspond with the bias-free angular rates, supplied by the ground-truth, and the bias-corrected angular rates of the automotive-grade IMU.

Substituting (24) and (26) in (28) yields

(29) $$\begin{array}{c} \left(\begin{array}{c} f_{im,x}^{g}-{\tilde{f}}_{im,x}^{m}+{\tilde{f}}_{im,x\;0}^{m}-f_{im,x\;0}^{g}\\ f_{im,y}^{g}-{\tilde{f}}_{im,y}^{m}+{\tilde{f}}_{im,y\;0}^{m}-f_{im,y\;0}^{g}\\ f_{im,z}^{g}-{\tilde{f}}_{im,z}^{m}+{\tilde{f}}_{im,z\;0}^{m}-f_{im,z\;0}^{g} \end{array}\right)=\left(\begin{array}{ccc} 0 & -{\tilde{f}}_{im,z}^{m}+f_{im,z\;0}^{g} & {\tilde{f}}_{im,y}^{m}-f_{im,y\;0}^{g}\\ {\tilde{f}}_{im,z}^{m}-f_{im,z\;0}^{g} & 0 & -{\tilde{f}}_{im,x}^{m}+f_{im,x\;0}^{g}\\ -{\tilde{f}}_{im,y}^{m}+f_{im,y\;0}^{g} & {\tilde{f}}_{im,x}^{m}-f_{im,x\;0}^{g} & 0 \end{array}\right)\left(\begin{array}{c} \phi_{mg}\\ \theta_{mg}\\ \psi_{mg} \end{array}\right), \end{array}$$

where the terms corresponding to the multiplication of the biases and misalignment angles have been assessed to be negligible.

The combination of (27) and (29) leads to

(30) $$\begin{array}{c} \left(\begin{array}{c} \omega_{x}^{g}-\omega_{x}^{m}\\ \omega_{y}^{g}-\omega_{y}^{m}\\ \omega_{z}^{g}-\omega_{z}^{m}\\ f_{im,x}^{g}-{\tilde{f}}_{im,x}^{m}+{\tilde{f}}_{im,x\;0}^{m}-f_{im,x\;0}^{g}\\ f_{im,y}^{g}-{\tilde{f}}_{im,y}^{m}+{\tilde{f}}_{im,y\;0}^{m}-f_{im,y\;0}^{g}\\ f_{im,z}^{g}-{\tilde{f}}_{im,z}^{m}+{\tilde{f}}_{im,z\;0}^{m}-f_{im,z\;0}^{g} \end{array}\right)=\left(\begin{array}{ccc} 0 & -\omega_{z}^{m} & \omega_{y}^{m}\\ \omega_{z}^{m} & 0 & -\omega_{x}^{m}\\ -\omega_{y}^{m} & \omega_{x}^{m} & 0\\ 0 & -{\tilde{f}}_{im,z}^{m}+f_{im,z\;0}^{g} & {\tilde{f}}_{im,y}^{m}-f_{im,y\;0}^{g}\\ {\tilde{f}}_{im,z}^{m}-f_{im,z\;0}^{g} & 0 & -{\tilde{f}}_{im,x}^{m}+f_{im,x\;0}^{g}\\ -{\tilde{f}}_{im,y}^{m}+f_{im,y\;0}^{g} & {\tilde{f}}_{im,x}^{m}-f_{im,x\;0}^{g} & 0 \end{array}\right)\left(\begin{array}{c} \phi_{mg}\\ \theta_{mg}\\ \psi_{mg} \end{array}\right), \end{array}$$

which may be treated as a linear identification problem of the following form:

(31) $$y_{k}=H_{k}\;\rho +w_{k},$$

where $w_{k}$ represents the measurement noise, which is assumed to be Gaussian, white, zero-mean and independent with a constant covariance matrix *R*. Please note that the first three diagonal elements of the measurement noise covariance *R* are directly linked to the ground-truth and IMU gyro noises, while the last three elements relate to the noise characteristics of the accelerometers.

The well-know recursive least squares (RLS) algorithm represents a logical choice to solve this identification problem. Exponential convergence in estimation algorithms is a relevant property since it generally leads to enhanced robustness against noise and modelling errors, as well as an improved performance in the non-stationary case [32]. The integration of an exponential forgetting factor λ∈(0,1) in the RLS algorithm is necessary to achieve exponential convergence and, additionally, it prevents the loss of estimation capability  [33]. Therefore, the incorporation of an exponential forgetting factor in the RLS algorithm is common practice. Nevertheless, a sufficient excitation property is usually required to guarantee exponential convergence.

**Definition** **1.** *The sequence $H_{k}$ is persistently exciting if there exist an integer h>0 and a real constant α>0 such that, for all integer k≥0*

(32) $$\sum_{s=0}^{h-1}H_{k}\;H_{k}^{T}\geq \alpha \;I.$$

If the sequence $H_{k}$ is persistently exciting, the estimates of the RLS with forgetting factor converge exponentially to the true parameters [33]. However, if the excitation is poor, the estimation problem becomes ill-conditioned, leading to unreliable estimates strongly affected by noise and model uncertainties.

In order to increase the robustness of the estimator against periods of poor excitation, a regularised recursive least squares algorithm with exponential forgetting factor is used for the identification of the misalignment between the ground-truth and the IMU (see Algorithm 1). **Algorithm 1:** Regularised recursive least squares [34].1k=k+1**input**: $H_{k}$, $P_{k|k-1}$, λ, $\Sigma_{R}$2$K_{k}=P_{k|k-1}\;H_{k}^{T}\;\left(R+H_{k}\;P_{k|k-1}\;H_{k}^{T}\right)$*%Gain as in RLS*3$P_{k|k}=P_{k|k-1}+K_{k}\;H_{k}\;P_{k|k-1}$*%Covariance update as in RLS*4${\bar{P}}_{k+1|k}=\frac{1}{\lambda }\;P_{k|k}$*%Covariance after forgetting*5$P_{k+1|k}={\bar{P}}_{k+1|k}\;{\left[I+\left(1-\lambda \right)\;\Sigma_{R}\;{\bar{P}}_{k+1|k}\right]}^{-1}$*%Covariance after regularization*6${\hat{\rho }}_{k}={\hat{\rho }}_{k-1}+K_{k}\;\left(y_{k}-H_{k}\;\rho_{k-1}\right)$*%Update parameter estimate***output**: ${\hat{\rho }}_{k}$, $P_{k+1|k}$

The suggested calibration manoeuvre is a figure eight drive since it provides a high level of excitation and stimulates the individual IMU elements equally in both directions. However, other manoeuvres that satisfy the persistent excitation condition of Defination 1 may also lead to satisfactory estimation results.

### 4.2. Experimental Validation

The performance of the algorithm is evaluated using data collected from figure eight drives. The ground-truth angular rates and specific forces are supplied by the INS/GNSS deeply coupled measurement system. This accurate equipment contains a high-precision IMU with MEMS accelerometers and fibre optic gyros (FOG), with biases specified as low as $0.01\;m/s^{2}$ and 1deg/h, respectively. As for the IMU angular rates and specific forces, they are obtained from a significantly less accurate automotive-grade IMU.

Please note that the real misalignment between the INS/GNSS system and the automotive-grade IMU is not known. Hence, the efficacy of the algorithm is analysed based on the following three criteria:

- The algorithm ability to follow artificially introduced misalignment errors.
- The misalignment estimation robustness against noise.
- The consistency of the misalignment estimates obtained from a set of different measurements.

The algorithm ability to track the misalignment angles can be illustrated by introducing artificial misalignment errors in the original data and validate whether these errors are tracked. As for the second aspect, the addition of different noise sequences with similar characteristics as the original measurement noise should not imply a significant change in the estimation results. Finally, since the misalignment angles do not change as long as the equipment is not unmounted, the variability of the estimates obtained from datasets collected during manoeuvres performed at different points in time should be low.

**Figure 9:**
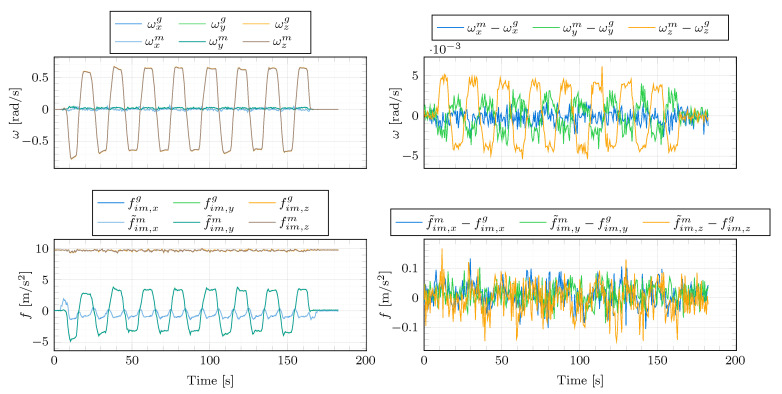
Measurements during a figure eight manoeuvre. The ground-truth angular rates ($\omega_{x}^{g}$, $\omega_{y}^{g}$, $\omega_{z}^{g}$) and specific forces ($f_{im,x}^{g}$, $f_{im,y}^{g}$, $f_{im,z}^{g}$) obtained from the high-performance INS/GNSS system and transformed to the mounting position of the automotive-grade IMU, and the bias-corrected angular rates ($\omega_{x}^{m}$, $\omega_{y}^{m}$, $\omega_{z}^{m}$) and bias-corrupted specific forces (${\tilde{f}}_{im,x}^{m}$, ${\tilde{f}}_{im,y}^{m}$, ${\tilde{f}}_{im,z}^{m}$) supplied by the automotive-grade IMU.

**Figure 10:**
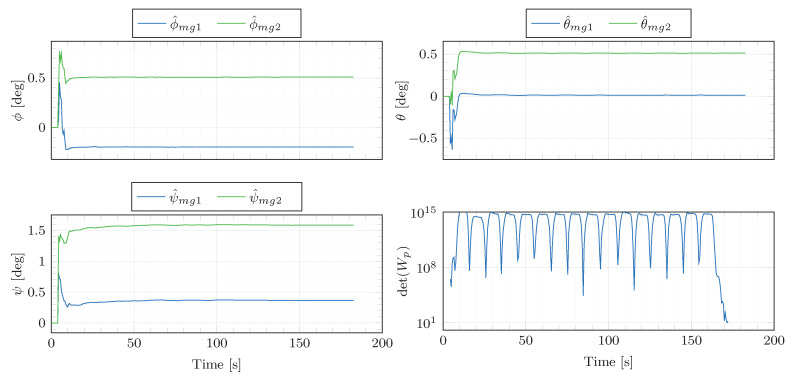
Estimation results for the figure eight manoeuvre of Figure 9. ${\hat{\phi }}_{mg1}$, ${\hat{\theta }}_{mg1}$, ${\hat{\psi }}_{mg1}$ are the parameter estimates obtained based on the original data. ${\hat{\phi }}_{mg2}$, ${\hat{\theta }}_{mg2}$, ${\hat{\psi }}_{mg2}$ are the parameter estimates obtained based on the data corrupted by the artificial misalignment error (0.7,0.5,1.2)deg for $\phi_{mg}$, $\theta_{mg}$ and $\psi_{mg}$, respectively. Finally, the determinant of the excitation matrix $W_{p}=H_{k}\;H_{k}^{T}$ is depicted in the last sub-plot.

The angular rate and specific force measurements supplied by the automotive-grade IMU and the high-performance INS/GNSS system are displayed in Figure 9. Table 2 outlines the parameters from Algorithm 1 employed to produce the presented experimental results. The misalignment estimates are presented in Figure 10. The figure displays the results based on the original data (${\hat{\phi }}_{mg1}$, ${\hat{\theta }}_{mg1}$, ${\hat{\psi }}_{mg1}$) as well as the estimates obtained from the data corrupted by artificially added misalignment errors (${\hat{\phi }}_{mg2}$, ${\hat{\theta }}_{mg2}$, ${\hat{\psi }}_{mg2}$). The artificially added errors are 0.7deg for $\phi_{mg}$, 0.5deg for $\theta_{mg}$ and 1.2deg for $\psi_{mg}$. Additionally, the IMU specific force signals are corrupted by artificial accelerometer biases of $\left(b_{f_{x}},b_{f_{y}},b_{f_{z}}\right)=\left(0.1,-0.1,-0.2\right)\;m/s^{2}$. This aims at showing that the misalignment angles are correctly estimated despite the accelerometer biases. As shown in Figure 10, the estimator is able to track the artificially added misalignment errors despite the added accelerometer biases, presenting increments between the estimates obtained from the misalignment-non-corrupted and misalignment-corrupted data of 0.71, 0.50 and 1.22 deg.

**Figure 11:**
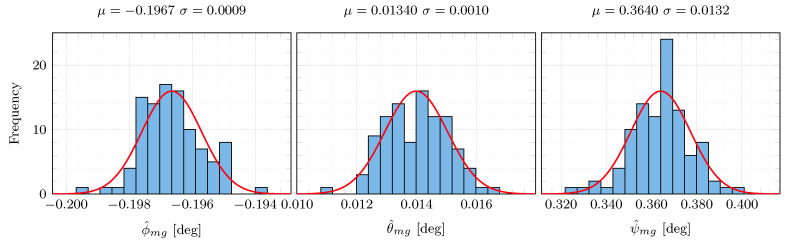
Estimation robustness against noise. One hundred different sequences of additive Gaussian white noises with standard deviations 0.001rad/s and $0.05\;m/s^{2}$ have been added to the original IMU angular rate and specific force data, respectively. The estimator has been run for each sequence. The last estimated values of each run have been collected in these histograms. The red curves represent the corresponding best Gaussian distribution fits. The corresponding mean μ and standard deviation σ are added on top of each plot.

Furthermore, the determinant of the excitation matrix $W_{p,k}=H_{k}\;H_{k}^{T}$ is far from zero, which experimentally shows that the persistence excitation condition is satisfied and the estimation problem is well-conditioned. This is confirmed by the analysis of the robustness against noise (see Figure 11). The original IMU angular rates and specific forces have been corrupted by 100 different sequences of additive Gaussian white noise with standard deviation σ=0.001rad/s and σ=0.05$m/s^{2}$, respectively. One can see that the misalignment estimates remain consistent despite the use of different additive noise sequences, which shows the algorithm robustness against noise. This claim is also supported by the variability of the misalignment estimates obtained from a set of different figure eight manoeuvres (Table 3). The estimation results are consistent throughout the different datasets, presenting the maximum differences in the estimated ${\hat{\phi }}_{mg}$, ${\hat{\theta }}_{mg}$ and ${\hat{\psi }}_{mg}$ of 0.008, 0.007 and 0.067deg, respectively.

## 5. IMU–Vehicle Misalignment Estimation

This section presents the main contribution of the paper, i.e., a method for the estimation of all three IMU–vehicle misalignment angles, which relies on series chassis sensors, an automotive-grade IMU and a single-track model.

**Figure 12:**
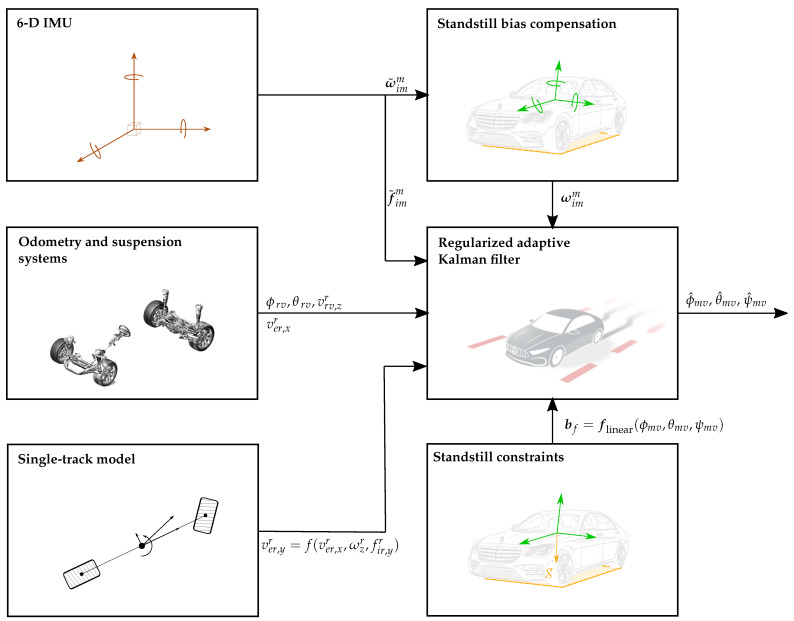
Estimation approach for the IMU–vehicle misalignment estimation. The proposed regularized adaptive Kalman filter relies on the specific forces (${\tilde{f}}_{im}^{m}$) supplied by an automotive-grade 6D IMU, its bias-compensated angular rates ($\omega_{im}^{m}$) as well as over-road longitudinal, lateral and vertical velocity information ($v_{er,x}^{r}$, $v_{er,y}^{r}$, $v_{rv,z}^{r}$) mainly provided by the odometry, single-track model and suspension, respectively. Furthermore, the vehicle-to-road orientation ($\phi_{rv}$, $\theta_{rv}$), computed from the suspension signals, is used to relate the over-road velocities to the vehicle velocity ($v_{ev}^{v}$), see Figure 4. Finally, constraints stemming from a standstill phase are used to enhance the performance of the estimator.

On the one hand, the IMU specific forces and angular rates are motion variables expressed in the IMU coordinate system (*m*). On the other hand, the information stemming from the odometry, suspension and single-track model constitutes a 3D velocity information source, which can be expressed in the defined vehicle coordinate system (*v*). Unlike for the ground-truth–IMU (*g*–*m*) and ground-truth–vehicle (*g*–*v*) extrinsic calibration, there is not a direct correlation between the measurements expressed in the IMU coordinate system (*m*) and the information represented in the vehicle coordinate system (*v*). Note that the specific forces and angular rates are not directly related to the velocities but to their time derivatives. This entails a significant increase in complexity, for which the previous approaches are no longer suitable. Notwithstanding this, the IMU–vehicle extrinsic calibration can be addressed as a joint parameter and state estimation problem. An adaptive estimator, i.e., the regularized adaptive Kalman filter, is proposed to estimate the vehicle velocities and attitude angles (states) as well as the IMU accelerometer biases and the IMU–vehicle misalignment angles (parameters). Furthermore, in order to bolster the performance of the estimator, additional standstill information is included in the form of constraints, see Figure 12.

The rest of this section is organized as follows. First, the model is derived, upon which the adaptive estimator is built. Afterwards, the estimator design is thoroughly described and the conditions that guarantee the convergence of the estimates are discussed. Finally, the validity of the proposed calibration procedure is experimentally exemplified.

### 5.1. Model

As in the case of the ground-truth–IMU (*g*–*m*) misalignment estimation, when addressing the IMU–vehicle extrinsic calibration, the IMU biases must be taken into account. Hence, the estimation problem of the IMU–vehicle misalignment also extends to that of estimating both the IMU biases and the misalignment angles. Please note that, in the absence of sensor faults and drastic changes in the environment, the biases remain nearly constant within intervals of few minutes. Furthermore, since the angular rate biases can easily be determined during standstill phases, the challenge is to estimate the accelerometer biases together with the misalignment angles.

In the derivation of the system equations, the following considerations are taken into account:

- Misalignment angles and IMU biases are small, which implies that second-order products involving these variables are negligible.
- Bearing in mind that the car angular motion remains small, particularly for pitch and roll movements, $\alpha \;{\dot{\phi }}_{\;rv}$ and $\alpha \;{\dot{\theta }}_{\;rv}$ may be neglected (where α represents a small angle).
- The misalignment angles remain within the range of [−3,3]deg and, hence, the small-angle approximation holds (sinα≈α, cosα≈1).

Keeping in mind that the vehicle and IMU coordinate systems are not aligned, the kinematic differential equations based on the 6D IMU signals are expressed in the IMU coordinate system [3]:

(33) $$\begin{array}{c} \begin{array}{cc} {\dot{x}}_{1} & =\omega_{\;y}^{m}\;x_{6}-\omega_{\;z}^{m}\;x_{2}\\ {\dot{x}}_{2} & =\omega_{\;x}^{m}\;x_{6}+\omega_{\;z}^{m}\;x_{1}\\ {\dot{x}}_{3} & =\omega_{\;z}^{m}\;x_{4}-\omega_{\;y}^{m}\;x_{5}+g\;x_{1}+{\tilde{f}}_{iv,x}^{m}-b_{f,x}^{m}\\ {\dot{x}}_{4} & =-\omega_{\;z}^{m}\;x_{3}+\omega_{\;x}^{m}\;x_{5}-g\;x_{2}+{\tilde{f}}_{iv,y}^{m}-b_{f,y}^{m}\\ {\dot{x}}_{5} & =\omega_{\;y}^{m}\;x_{3}-\omega_{\;x}^{m}\;x_{4}-g\;x_{6}+{\tilde{f}}_{iv,z}^{m}-b_{f,z}^{m}\\ {\dot{x}}_{6} & =-\omega_{\;y}^{m}\;x_{1}-\omega_{\;x}^{m}\;x_{2}, \end{array} \end{array}$$

where

(34) $$\begin{array}{cc} x & ={\left(\begin{array}{c} \sin\theta_{\;nm},\;\sin\phi_{\;nm}\;\cos\theta_{\;nm}\;,\;v_{ev,x}^{m}\;,\;v_{ev,y}^{m}\;,\;v_{ev,z}^{m}\;,\;\cos\theta_{\;nm}\;\cos\phi_{\;nm} \end{array}\right)}^{T} \end{array}$$

As in [3], considering the accuracy of automotive-grade IMUs, the rotation of the Earth as well as the transport rate are neglected. Note that $\omega^{m}=\omega_{iv}^{m}$ instead of $\omega_{nv}^{m}$ is used. Furthermore, the IMU specific force signals are transformed to the origin of the vehicle coordinate system, note that ${\tilde{f}}_{iv}^{m}$ instead of ${\tilde{f}}_{im}^{m}$ is used.

Additionally, using the IMU–vehicle misalignment angles ($\phi_{mv}$, $\theta_{mv}$, $\psi_{mv}$), the vehicle-to-road orientation ($\phi_{rv}$, $\theta_{rv}$), the single-track model, and the over-road longitudinal and vertical velocities ($v_{\;er,x}^{r}$, $v_{\;rv,z}^{r}$), the following measurement model is obtained (see Appendix A for a thorough derivation):

(35) $$\begin{array}{cc} y_{1}=\left[\begin{array}{c} {\tilde{v}}_{x}^{v}+h_{p}\;{\dot{\theta }}_{\;rv} \end{array}\right] & =v_{ev,x}^{m}+\psi_{mv}\left[{\tilde{v}}_{y}^{v}\right]-\theta_{mv}\left[{\tilde{v}}_{z}^{v}\right]\\ y_{2}=\left[\begin{array}{c} {\tilde{v}}_{y}^{v}-h_{r}\;{\dot{\theta }}_{\;rv} \end{array}\right] & =v_{ev,y}^{m}-\psi_{mv}\left[{\tilde{v}}_{x}^{v}+SG\;v_{\;er,x}^{r}\;{\tilde{f}}_{iv,x}^{m}\right]+\phi_{mv}\left[{\tilde{v}}_{z}^{v}+SG\;v_{\;er,x}^{r}\;\left({\tilde{f}}_{iv,z}^{m}+{\tilde{f}}_{iv,y}^{m}\;\phi_{\;rv}\right)\right]\\ & -\theta_{mv}\left[SG\;v_{\;er,x}^{r}\;\left({\tilde{f}}_{iv,z}^{m}\;\phi_{\;rv}\right)\right]-b_{f_{y}}\left[SG\;v_{\;er,x}^{r}\right]+b_{f_{z}}\left[SG\;v_{\;er,x}^{r}\;\phi_{\;rv}\right]\\ y_{3}=\left[{\tilde{v}}_{z}^{v}\right] & =v_{ev,z}^{m}+\theta_{mv}\left[{\tilde{v}}_{x}^{v}+SG\;v_{\;er,x}^{r}\;{\tilde{f}}_{iv,x}^{m}\;\phi_{\;rv}^{2}\right]\\ & -\phi_{mv}\left[{\tilde{v}}_{y}^{v}+SG\;v_{\;er,x}^{r}\;\left({\tilde{f}}_{iv,z}^{m}\;\phi_{\;rv}+{\tilde{f}}_{iv,y}^{m}\;\phi_{\;rv}^{2}\right)\right]+\psi_{mv}\left[SG\;v_{\;er,x}^{r}\;{\tilde{f}}_{iv,x}^{m}\;\phi_{\;rv}\right] \end{array}$$

where SG is the side-slip angle gradient, $h_{r}$ and $h_{p}$ are the height of the origin of the vehicle coordinate system with respect to the roll and pitch axes, and 

(36) $$\begin{array}{cc} {\tilde{v}}_{x}^{v} & =v_{\;er,x}^{r}-v_{\;rv,z}^{r}\;\theta_{\;rv} \end{array}$$

(37) $$\begin{array}{cc} {\tilde{v}}_{y}^{v} & =l_{r}\;\omega_{z}^{m}-SG\;v_{\;er,x}^{r}\;\left({\tilde{f}}_{iv,y}^{m}-{\tilde{f}}_{iv,z}^{m}\;\phi_{\;rv}\right)+v_{\;rv,z}^{r}\;\phi_{\;rv} \end{array}$$

(38) $$\begin{array}{cc} {\tilde{v}}_{z}^{v} & =v_{\;rv,z}^{r}+v_{\;er,x}^{r}\;\theta_{\;rv}-\left(l_{r}\;\omega_{z}^{m}-SG\;v_{\;er,x}^{r}\;\left({\tilde{f}}_{iv,y}^{m}-{\tilde{f}}_{iv,z}^{m}\;\phi_{\;rv}\right)\right)\;\phi_{\;rv} \end{array}$$

In (35), the terms in the square brackets ($\left[...\right]$) are known, and the unknown states and parameters are highlighted in blue and red, respectively.

Combining the differential equations in (33) with the measurement model (35), the system can be represented in a state-affine form as follows:

(39) $$\begin{array}{c} \left\{\begin{array}{cc} \dot{x} & =A(u)x+b(u)+\Phi \rho \\ y & =Cx+\Psi (y)\;\rho \end{array},\right. \end{array}$$

where

(40) $$\begin{array}{cc} x & ={\left(\sin\theta_{\;nm},\;\sin\phi_{\;nm}\;\cos\theta_{\;nm}\;,\;v_{ev,x}^{m}\;,\;v_{ev,y}^{m}\;,\;v_{ev,z}^{m}\;,\;\cos\theta_{\;nm}\;\cos\phi_{\;nm}\right)}^{T} \end{array}$$

(41) $$\begin{array}{cc} y & ={\left({\tilde{v}}_{x}^{v}+h_{p}\;{\dot{\theta }}_{\;rv},{\tilde{v}}_{y}^{v}-h_{r}\;{\dot{\theta }}_{\;rv},{\tilde{v}}_{z}^{v}\right)}^{T} \end{array}$$

(42) $$\begin{array}{cc} u & ={\left(\omega_{\;x}^{m},\omega_{\;y}^{m},\omega_{\;z}^{m},{\tilde{f}}_{iv,x}^{m},{\tilde{f}}_{iv,y}^{m},{\tilde{f}}_{iv,z}^{m}\right)}^{T} \end{array}$$

(43) $$\begin{array}{cc} \rho & ={\left(\phi_{mv},\theta_{mv},\psi_{mv},b_{f_{x}},b_{f_{y}},b_{f_{z}}\right)}^{T} \end{array}$$

### 5.2. Estimator Design

In this section, an adaptive estimator, i.e., the regularised adaptive Kalman filter [35,36], is proposed to address the extrinsic calibration between the IMU and vehicle coordinate systems. First, a discretisation is applied to transform the continuous-time system into a discrete-time system and, then, the adaptive estimator is designed based on the discretized system. Furthermore, the conditions that guarantee the convergence of the estimates are outlined. Finally, the integration of additional information beyond the one explicitly described in the system model is discussed. Specifically, the use of constraints determined during a standstill phase is proposed in order to improve the performance of the estimator.

#### 5.2.1. Discrete-Time Implementation

Most systems in the real world are characterized with continuous-time dynamics. Nevertheless, estimators are customarily run in digital computers and, hence, continuous-time system are frequently discretised. Bearing in mind that the car body motion commonly lies within the range of 1–2 Hz [37], 100 Hz is chosen as the sampling frequency. Assuming that the specific force and angular rates remain constant between samples, the exact discretisation of (39) may be obtained as described in [3]. However, in order to reduce the computational load, the discretisation may be simplified in practice by using a first-order approximation or a lookup table.

The resulting discretised system is presented below:

(44) $$\begin{array}{c} \left\{\begin{array}{c} x_{k}=A_{d}\left(u_{k}\right)\;x_{k-1}+b_{d}\left(u_{k}\right)+\Phi_{d}\left(u_{k}\right)\;\rho \\ y_{k}=C\;x_{k}+\Psi \left(y_{k}\right)\;\rho \end{array},\right. \end{array}$$

where $A_{d}$, $b_{d}$ and $\Phi_{d}$ are the matrices that describe the discrete-time system, and $x_{k}=x\left(t_{k}\right)$, $u_{k}=u\left(t_{k}\right)$ and $y_{k}=y\left(t_{k}\right)$.

Note that $u_{k}$ and $y_{k}$ are known at any $t_{k}$ and, therefore, the system can be considered as a discrete-time linear time-varying system [38]:

(45) $$\begin{array}{c} \left\{\begin{array}{c} x_{k}=A_{d,k}\;x_{k-1}+b_{d,k}+\Phi_{d,k}\;\rho \\ y_{k}=C\;x_{k}+\Psi_{k}\;\rho \end{array}\right. \end{array}$$

#### 5.2.2. Algorithm Description

The proposed algorithm to estimate the misalignment between the IMU and the vehicle coordinate systems is built upon the discrete-time state-space representation in (45). On the one hand, the angular rates and specific forces supplied by the IMU are used for the state propagation, based on the kinematic differential equations from (33). On the other hand, the measurement model (35) uses the IMU–vehicle misalignment in order to relate the information provided by the over-road longitudinal and vertical velocities, as well as the single-track model to the propagated velocities. The resulting discrete-time system is represented in a state-affine form, allowing thereby the application of the regularised adaptive Kalman filter.

The structure of the regularised adaptive Kalman filter can be broken down into two parts. First, the algorithm is built upon a linear Kalman filter, which carries out the estimation of the states assuming known parameters. Second, the estimator is bolstered by an RLS-based adaptation law, which addresses the estimation of the parameters. The algorithm is shown in Algorithm 2. The state propagation, (a.1)–(a.2), is equivalent to that of the linear Kalman filter. In this part, the IMU signals are used to compute the predicted state estimates. The computation of the Kalman gain and updated error covariance takes place in (a.3)–(a.4), which is identical to the Kalman filter. The RLS-based adaptation law can be found in (a.5)–(a.13), where the gain $\Gamma_{k}$ for the parameter correction is computed. Finally, the state and parameter estimates are updated based on the measured signals in (a.14)–(a.16). Then, the recursion repeats using the next measurement. **Algorithm 2** Regularised Adaptive Kalman Filter (RAKF)**INITIALIZATION****1**$P_{0}^{+}=P_{0}$; $\Upsilon_{0}=0$; $S_{0|0}=S_{1|0}=S_{0}$; ${\hat{x}}_{0|0}={\hat{x}}_{0}$; ${\hat{\rho }}_{0}={\hat{\rho }}_{0}$**RECURSION****2**k=k+1Prediction**3**${\hat{x}}_{k|k-1}=A_{k}\;{\hat{x}}_{k-1|k-1}+B_{k}\;u_{k}+\Phi_{k}\;{\hat{\rho }}_{k-1}$*%State prediction as in the KF*(a.1)**4**$P_{k|k-1}=A_{k}\;P_{k-1|k-1}\;A_{k}^{T}+Q_{k}$*%Covariance prediction as in the KF*(a.2)**Innovation****5**$\Sigma_{k}=C_{k}\;P_{k|k-1}\;C_{k}^{T}+R_{k}$*%Predicted measurement covariance as in the KF*(a.3)**6**$K_{k}=P_{k|k-1}\;C_{k}^{T}\;\Sigma_{k}^{-1}$*%Kalman Gain as in the KF*(a.4)**7**$P_{k|k}=\left[I_{n}-K_{k}\;C_{k}\right]\;P_{k|k-1}$*%Covariance innovation as in KF*(a.5)**8**$\Omega_{k}=C_{k}\;A_{k}\;\Upsilon_{k-1}+C_{k}\;\Phi_{k}+\Psi_{k}$*%Aux. var. (represents excitation for parameter estimation)*(a.6)**9**$\Lambda_{k}={\left[\Sigma_{k}+\Omega_{k}\;S_{k|k-1}\Omega_{k}^{T}\right]}^{-1}$*%Var. for simplification of* (a.8) *and* (a.9)(a.7)**10**$\Gamma_{k}=S_{k|k-1}\;\Omega_{k}^{T}\;\Lambda_{k}$*%Parameter gain as in RLS*(a.8)**11**$S_{k|k}=S_{k|k-1}-S_{k|k-1}\;\Omega_{k}^{T}\Lambda_{k}\;\Omega_{k}\;S_{k|k-1}$*%Parameter covariance update as in the RLS*(a.9)**12**${\bar{S}}_{k+1|k}=\frac{1}{\lambda }\;S_{k|k}$*%Parameter covariance after forgetting*(a.10)**13**$S_{k+1|k}={\bar{S}}_{k+1|k}\;{\left[I+\left(1-\lambda \right)\;\Sigma_{R}\;{\bar{S}}_{k+1|k}\right]}^{-1}$*%Parameter covariance after regularization*(a.11)**14**$\Upsilon_{k}=\left[I-K_{k}\;C_{k}\right]\;A_{k}\;\Upsilon_{k-1}+\left[I-K_{k}\;C_{k}\right]\;\Phi_{k}-K_{k}\;\Psi_{k}$*%Aux. var.*(a.12)**15**${\tilde{y}}_{k}=y_{k}-C_{k}\;{\hat{x}}_{k|k-1}-\Psi_{k}\;{\hat{\rho }}_{k-1}$*%Measurement error (meas-pred)*(a.13)**16**$\Delta \rho_{k}=\Gamma_{k}\;{\tilde{y}}_{k}$*%Parameter correction*(a.14)**17**${\hat{\rho }}_{k}={\hat{\rho }}_{k-1}+\Delta \rho_{k}$*%Parameter update*(a.15)**18**${\hat{x}}_{k|k}={\hat{x}}_{k|k-1}+K_{k}\;{\tilde{y}}_{k}+\Upsilon_{k}\;\Delta \rho_{k}$*%State update*(a.16)${\hat{x}}_{k|k-1}$, ${\hat{x}}_{k|k}$: A priori and a posteriori state estimates.$P_{k|k-1}$, $P_{k|k}$: A priori and a posteriori state error covariance matrices.


The exponential convergence of the state and parameter estimates is guaranteed provided that the $\left[A_{k},C_{k}\right]$ pair is uniformly completely observable, the $\left[A_{k},Q_{k}^{\frac{1}{2}}\right]$ pair is uniformly completely controllable [39], and the system is being persistently excited:

**Definition** **2.** *The matrices $A_{k}$, $C_{k}$, $\Phi_{k}$ and $\Psi_{k}$ are persistently exciting if there exist an integer h>0 and a real constant α>0 such that, for all integer j≥0, the matrix sequence $\Omega_{k}$ driven by $A_{k}$, $C_{k}$, $\Phi_{k}$ and $\Psi_{k}$ through the linear system* (a.6) *and* (a.12)*, satisfies*

(46) $$\sum_{k=j+1}^{j+h}W_{p,k}\geq \alpha \;I,\;\mathrm{where}\;W_{p,k}≜\Omega_{k}^{T}\;\Sigma_{k}^{-1}\;\Omega_{k}$$

The regularization algorithm integrated in the adaptation law (a.10) enhances the robustness of the estimator so that, despite poor excitation, acceptable estimates are supplied. More precisely, it prevents the so-called covariance wind-up phenomenon. During phases of poor excitation, when the information is insufficient to determine all parameters, some elements of $S_{k|k}$ tend to grow unlimited, which may lead to an aggressive behaviour and large estimation errors. The regularization algorithm sets bounds to this growth while keeping the exponential convergence properties of the system when excitation is available. This results in a more reliable and accurate estimation of both states and parameters.

In [3], it was already shown that, assuming known parameters, the system is always observable. Hence, the $\left[A_{k},C_{k}\right]$ pair is uniformly completely observable. Furthermore, it was also shown that, since $Q_{k}$ is a positive definite matrix, the $\left[A_{k},Q_{k}^{\frac{1}{2}}\right]$ pair is uniformly completely controllable. In spite of regularization, sufficient excitation is required for the parameter convergence. Ensuring that, during a calibration manoeuvre, the persistent excitation condition is met guarantees the convergence of the parameter estimates. This may easily be investigated by computing the excitation matrix $W_{p,k}$ online and analysing how close it is to singularity. For this purpose, the determinant is used. A $\det(W_{p,k})$ close to zero indicates that the excitation is poor while large values of $\det(W_{p,k})$ imply that the information is sufficient to estimate all the parameters.

#### 5.2.3. Estimator with Constraints

The specific force during standstill corresponds with the reaction to the acceleration due to gravity:

(47) $$\begin{array}{c} f_{iv}^{v}=-g^{v} \end{array}$$

Hence, if the attitude angles (pitch and roll) of the vehicle coordinate system are known, the values of each specific force component can be deduced. This relationship can be exploited during the calibration procedure to incorporate additional information to the estimator. Before or after the calibration manoeuvre, the vehicle could remain stationary in an area where it is feasible to identify its attitude angles. An option would be to stand still on a horizontal surface (normal to gravity) and infer the specific force components in the vehicle coordinate system from the vehicle levels:

(48) $$\begin{array}{cc} f_{iv,x\;0}^{v} & =-g\;\sin\theta_{\;rv}\\ f_{iv,y\;0}^{v} & =g\;\sin\phi_{\;rv}\;\cos\theta_{\;rv}\\ f_{iv,z\;0}^{v} & =g\;\cos\phi_{\;rv}\;\cos\theta_{\;rv} \end{array}$$

Another option could be to identify the attitude angles of the vehicle coordinate system using perception sensors.

This information yields a relationship between biases and misalignment angles:

(49) $$\begin{array}{c} \left(\begin{array}{c} b_{f_{x}}\\ b_{f_{y}}\\ b_{f_{z}} \end{array}\right)=\left(\begin{array}{c} {\tilde{f}}_{iv,x\;0}^{m}\\ {\tilde{f}}_{iv,y\;0}^{m}\\ {\tilde{f}}_{iv,z\;0}^{m} \end{array}\right)-\left(\begin{array}{c} f_{iv,x\;0}^{v}\\ f_{iv,y\;0}^{v}\\ f_{iv,z\;0}^{v} \end{array}\right)+\left(\begin{array}{ccc} 0 & -f_{iv,z\;0}^{v} & f_{iv,y\;0}^{v}\\ f_{iv,z\;0}^{v} & 0 & -f_{iv,x\;0}^{v}\\ -f_{iv,y\;0}^{v} & f_{iv,x\;0}^{v} & 0 \end{array}\right)\;\left(\begin{array}{c} \phi_{mv}\\ \theta_{mv}\\ \psi_{mv} \end{array}\right), \end{array}$$

which can be integrated in the estimator in the form of constraints to improve the estimation performance. One way of doing this is to integrate the parameter equality constraint as an artificial measurement [40]:

(50) $$\begin{array}{cc} y_{4} & ={\tilde{f}}_{iv,x\;0}^{m}-f_{iv,x\;0}^{v}=f_{iv,z\;0}^{v}\;\theta_{mv}-f_{iv,y\;0}^{v}\;\psi_{mv}+b_{f_{x}}\\ y_{5} & ={\tilde{f}}_{iv,y\;0}^{m}-f_{iv,y\;0}^{v}=-f_{iv,z\;0}^{v}\;\phi_{mv}+f_{iv,x\;0}^{v}\;\psi_{mv}+b_{f_{y}}\\ y_{6} & ={\tilde{f}}_{iv,z\;0}^{m}-f_{iv,z\;0}^{v}=f_{iv,y\;0}^{v}\;\phi_{mv}-f_{iv,x\;0}^{v}\;\theta_{mv}+b_{f_{z}} \end{array}$$

Please note that these additional equations are affine with respect to the parameters. Hence, their integration in the system will not distort the system structure of (45). The resulting system will therefore take the following form:

(51) $$\begin{array}{c} \left\{\begin{array}{c} x_{k}=A_{d,k}\;x_{k-1}+b_{d,k}+\Phi_{d,k}\;\rho \\ y_{c,k}=C_{c}\;x_{k}+\Psi_{c,k}\;\rho \end{array},\right. \end{array}$$

where $y_{c,k}=\left({\tilde{v}}_{x,k}^{v}+h_{p}\;{\dot{\theta }}_{\;rv,k}\;,\;{\tilde{v}}_{y,k}^{v}-h_{r}\;{\dot{\theta }}_{\;rv,k}\;,\;{\tilde{v}}_{z,k}^{v}\;,\;y_{4,k}\;,\;y_{5,k}\;,\;y_{6,k}\right)$.

### 5.3. Experimental Results

In order to evaluate the performance of the described algorithm, an experimental validation is conducted. The test vehicle is a rear-wheel drive car equipped with the ground-truth system (described in Section 1.2), an automotive-grade MEMS 6D IMU and series chassis sensors. The analysed data have been generated during figure eight manoeuvres, which provide a high level of excitation and stimulate the individual axes equally in both directions. Unlike for the ground-truth–IMU relative orientation estimation, a restriction for the maximum lateral excitation is set in the execution of the manoeuvre so that the vehicle remains within the validity region of the single-track model ($|{\tilde{f}}_{iv,y}^{m}|⪅4\;\frac{m}{s^{2}}$).

Two different estimator designs are compared:

(A) The regularized adaptive Kalman filter (Algorithm 2) with artificial measurement constraints, according to (51).
(B) The regularized adaptive Kalman filter (Algorithm 2) without constraints, according to (45).

The aim is to illustrate the benefits of exploiting the additional standstill information, which is not explicitly given by the original system model.

Regarding the parametrisation of the estimator, the choice of the regularized adaptive Kalman filter parameters (Algorithm 2) is shown in Table 4. The parametrisation was selected based on the sensor specifications as well as data-driven evaluations of the proposed adaptive estimator. Moreover, a first-order approximation has been used to obtain the discretised system.

In order to validate the results of the misalignment estimates, these are compared against the misalignment angles obtained from the ground-truth–IMU and –vehicle extrinsic calibration, shown in Table 5.

**Figure 13:**
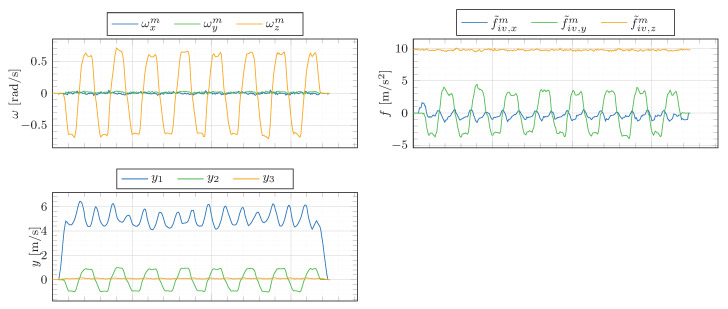
Estimator inputs during a figure eight drive. The bias-free angular velocities ($\omega_{x}$, $\omega_{y}$, $\omega_{z}$), the uncorrected specific forces (${\tilde{f}}_{iv,x}$, ${\tilde{f}}_{iv,y}$, ${\tilde{f}}_{iv,z}$), the measurements ($y_{1}$, $y_{2}$, $y_{3}$).

**Figure 14:**
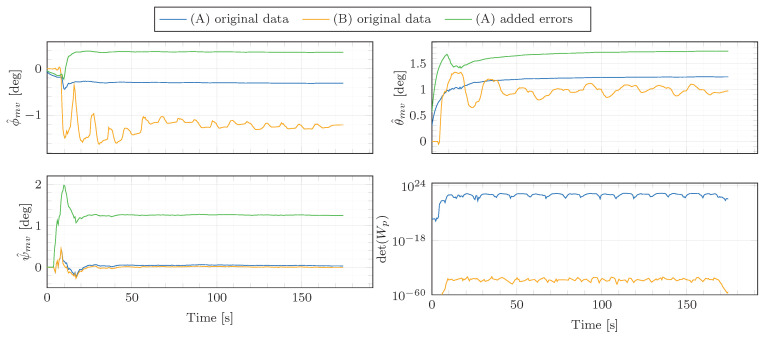
Estimation results during the figure eight manoeuvre of Figure 13 for both estimators (A) and (B). Additionally, in green, the parameter estimates obtained with estimator (A) based on the data corrupted by the artificial misalignment error (0.7,0.5,1.2)deg for $\phi_{mv}$, $\theta_{mv}$ and $\psi_{mv}$, respectively, and the artificial bias errors $\left(0.1,0.1,-0.2\right)\;m/s^{2}$ for $b_{f_{x}}$, $b_{f_{y}}$ and $b_{f_{z}}$, respectively. Finally, the determinant of the excitation matrix $W_{p,k}=\Omega_{k}^{T}\;\Sigma_{k}^{-1}\;\Omega_{k}$ is depicted in the last sub-plot.

The inputs of the estimator are depicted in Figure 13. Please note that the plotted angular rates ($\omega_{x}$, $\omega_{y}$, $\omega_{z}$) are bias-compensated. Furthermore, note that the lateral specific force mostly remains within the region of $|{\tilde{f}}_{iv,y}^{m}|<4\;\frac{m}{s^{2}}$. The corresponding estimation results are shown in Figure 14.

First, let us focus on the estimator without constraints, i.e., (B). Especially conspicuous in the plot is the fact that the determinant of the persistence excitation matrix is close to zero for this estimator (see last sub-plot), indicating that there is not sufficient information in order to properly infer all parameters. This may explain the unsteady behaviour of the misalignment estimates. Furthermore, the parameter estimates do not converge to the values computed from the ground-truth–IMU and –vehicle extrinsic calibration from Table 5. Hence, one can conclude that the outputs of (45) do not carry sufficient information to reach a satisfactory estimation of all the parameters, additional knowledge is required. In view of these results, estimator (B) is not considered in further analyses.

Things look different for the estimator with constraints, i.e., (A). Particularly noteworthy is the determinant of the persistence excitation matrix, which is not close to zero any more. This indicates that the estimator has enough information to properly infer all parameters. This may well be the reason for the steady behaviour exhibited by the parameter estimates, which stands in stark contrast with the results of the estimator without constraints, i.e., (B). As for the comparison of the results with respect to the values computed from the ground-truth–IMU and –vehicle extrinsic calibration (Table 5), the final estimated parameters are fairly close. The differences in $\phi_{mv}$, $\theta_{mv}$ and $\psi_{mv}$ are as low as 0.004, 0.056 and 0.067 deg, respectively. Hence, one can conclude that the integration of the additional standstill information in the form of constraints has improved the estimation results by making the system persistently excited during the calibration manoeuvre.

Additionally, in order to assess the ability of (A) to follow artificially introduced misalignments, the algorithm has also been run on corrupted data, where misalignments of (0.7,0.5,1.2)deg and accelerometer biases of $\left(0.1,-0.1,-0.2\right)\;m/s^{2}$ have been added to the original data. For the corrupted dataset, the misalignment estimates increased by 0.670, 0.503 and 1.212 deg for $\phi_{mv}$, $\theta_{mv}$ and $\psi_{mv}$, respectively. Hence, the results show that, despite the artificial biases, the algorithm is able to track the added misalignments.

Finally, the estimator (A) has been run using two additional data sets collected on different days: a less dynamic figure eight drive with $|{\tilde{f}}_{y\;\max}^{m}|\approx 3$$\frac{m}{s^{2}}$ (M2) and a more dynamic one in which $|{\tilde{f}}_{y\;\max}^{m}|\approx 5$$\frac{m}{s^{2}}$ (M3). This aims at analysing the variability of the misalignment estimates within a collection of different figure eight drives and, moreover, at assessing the effect of moderate deviations in the manoeuvre execution. The resulting misalignment estimates from these manoeuvres together with the ones obtained from the manoeuvre in Figure 13 (M1) are displayed in Table 6. The maximum differences are as low as 0.035, 0.0530 and 0.021deg for $\phi_{mv}$, $\theta_{mv}$ and $\psi_{mv}$, respectively. Furthermore, all results remain very close to the misalignments obtained from the m−g and v−g extrinsic calibration. Hence, one concludes that the estimation results remain consistent in spite of variations in the manoeuvre execution.

## 6. Conclusions

The present paper addresses the extrinsic calibration of a vehicle equipped with series chassis sensors and an automotive-grade IMU. Specifically, it proposes a method to estimate the misalignment between the IMU and vehicle coordinate systems. Unfortunately, due to tolerances in the manufacturing processes, the transformation between the IMU and vehicle coordinate systems is not perfectly known—while, due to the low angular motion of land vehicles, the positioning errors may be neglected, and misalignment errors have a significant impact on the motion estimates.

One of the challenges that arises when evaluating the performance of such an extrinsic calibration procedure is the fact that there is no simple way to measure the real misalignment. In this work, a reference is proposed based on a high-precision INS/GNSS system, which is taken as a ground-truth for the motion variables. Two calibration procedures to estimate the 3D rotation between the ground-truth and both the IMU and vehicle coordinate systems have been described and experimentally validated. The combination of these two rotations yields a reference for the 3D rotation between the IMU and vehicle coordinate systems.

The IMU–vehicle misalignment estimation has been addressed as a joint state and parameter estimation problem represented in a state-affine form. The approach relies on measurements from odometry, suspension and an automotive-grade IMU fused in a regularised adaptive Kalman filter. It has been experimentally shown with figure eight drives that the sole use of odometry, suspension and a single-track model does not supply enough excitation to determine all states and parameters. Additional information is required. In order to improve this aspect, a standstill phase, in which the vehicle attitude is known, is used to incorporate further information in the form of artificial measurements. Experimental results show that integrating this additional information decisively improves the performance of the estimator. The persistence excitation condition is satisfied and the estimated misalignments are consistent with the results obtained in the ground-truth–IMU and –vehicle calibration procedures. Additionally, the estimator is able to follow artificial variations in the misalignment angles and the variability of the estimates remains low despite alterations in the manoeuvre execution.

The results presented in this article show that the proposed calibration procedure is a robust and industrially viable method for the IMU–vehicle misalignment estimation. Unlike other errors, such as the time-varying IMU biases, the misalignment between the IMU and the vehicle is unlikely to change over time. In a series product, not equipped with a ground-truth system, the proposed IMU–vehicle misalignment estimation may be integrated into the end-of-line calibration of the vehicle. The calibration manoeuvre can be standardized and driven manually or automatically at the end of the production process. Moreover, the same method may also be used as a re-calibration procedure in the case of structural modifications in the vehicle or its sensors, such as after replacing the IMU. The identified misalignment angles may then be incorporated as parameters in a vehicle motion estimation module, which, based on misalignment-compensated IMU signals, supplies estimates of motion quantities during operation. It is left to the vehicle motion estimation algorithm running online to account for time-varying errors, such as the IMU in-run biases or the biases induced by ageing. This topic takes up a central question in [3], in which a method for the simultaneous estimation of the vehicle motion states and IMU biases is proposed and experimentally validated.

The usefulness of the knowledge of the 3D rotation between the ground-truth and both the IMU and vehicle coordinate systems goes beyond that of supplying a reference for the IMU–vehicle misalignment estimates. These transformations are required for a consistent evaluation of motion estimation algorithms based on the ground-truth.

In the proposed calibration procedure, GNSS and perception sensor information have not been used. However, if the vehicle is equipped with cameras, lidars, radars or accurate GNSS receivers, their information may be integrated in the calibration procedure in order to enhance its performance. However, this would come at a cost. A considerable increase in complexity and quite some effort in guaranteeing the reliability of the information supplied by these additional sensors is to be expected.

One of the limitations of the proposed approach is the fact that not all IMU systematic error contributions are considered, such as scaling factor, non-orthogonality or nonlinearity error components. Certainly, if these contributions are significant, the accuracy of the misalignment estimates may degrade. In order to mitigate the effects of these error contributions, an IMU end-of-line calibration procedure should be conducted by the sensor supplier prior to the IMU assembly in the vehicle [41,42]. Furthermore, the specific orientation of the vehicle coordinate system within the vehicle body may slightly vary due to effects such as tire wear, load distribution or suspension warp. These uncertainties have been neglected in this work since significant changes are not expected in normal operation conditions (no faults nor extreme wear), which has also been supported by the analysed data. However, a thorough analysis on a relatively large vehicle fleet over a large time span could be carried out to investigate these effects. Conclusions may be drawn regarding the frequency in which a misalignment calibration is needed and the particular events that should trigger it. This may be the subject of future work.

## Figures and Tables

**Table 1:** Estimation results of the *v*–*g* misalignment obtained from three calibration procedures performed on different dates. Results expressed in degrees.

Parameter	M1	M2	M3	Mean
${\hat{\phi }}_{vg}$	0.100	0.128	0.122	0.117
${\hat{\theta }}_{vg}$	−1.178	−1.158	−1.177	−1.171
${\hat{\psi }}_{vg}$	0.265	0.292	0.320	0.292

**Table 2:** Parameter choice, units according to the International System of Units.

Parameter	Value
*R*	diag([2 × 10${}^{-6}$, 2 × 10${}^{-6}$, 2 × 10${}^{-6}$, 1 × 10${}^{-3}$, 1 × 10${}^{-3}$, 1 × 10${}^{-3}$])
$P_{0}$	diag([3.0625 × 10${}^{-4}$, 3.0625 × 10${}^{-4}$, 3.0625 × 10${}^{-4}$])
${\hat{\rho }}_{0}$	[0, 0, 0]
$\Sigma_{R}$	diag([3.2653 × 10${}^{3}$, 3.2653 × 10${}^{3}$, 3.2653 × 10${}^{3}$])
λ	0.9998

**Table 3:** Variability misalignment estimation with angular rates and specific forces. Results expressed in degrees.

Parameter	M1	M2	M3	M4	M5	M6	M7	Max. Diff.	Mean
${\hat{\phi }}_{mg}$	−0.187	−0.188	−0.193	−0.193	−0.191	−0.193	−0.195	0.008	−0.192
${\hat{\theta }}_{mg}$	0.011	0.012	0.006	0.006	0.007	0.008	0.005	0.007	0.009
${\hat{\psi }}_{mg}$	0.434	0.373	0.367	0.367	0.402	0.401	0.394	0.067	0.396

**Table 4:** Estimator scheme parameter choice, units according to the International System of Units.

Parameter	Value
*Q*	diag([$2\times 10^{-6},2\times 10^{-6},1\times 10^{-3},1\times 10^{-3},1\times 10^{-3},1\times 10^{-8}$])
*R* (A)	diag([$1\times 10^{-2},1\times 10^{-1},1\times 10^{-2},1\times 10^{-4},1\times 10^{-4},1\times 10^{-4}$])
*R* (B)	diag([$1\times 10^{-2},1\times 10^{-1},1\times 10^{-2}$])
$P_{0}$	diag([$1.2\times 10^{-3},1.2\times 10^{-3},4\times 10^{-3},4\times 10^{-3},4\times 10^{-3},1.2\times 10^{-3}$])
$S_{0}$	diag([$8\times 10^{-3},8\times 10^{-3},8\times 10^{-3},1\times 10^{-2},1\times 10^{-2},1\times 10^{-2}$])
${\hat{x}}_{0}$	${\left(0,0,0,0,0,1\right)}^{T}$
${\hat{\rho }}_{0}$	${\left(0,0,0,0,0,0\right)}^{T}$
$\Sigma_{R}$	diag([1250,1250,1250,125,125,125])
λ	0.9996

**Table 5:** Misalignment angles computed from the identified *m*-*g* and *v*-*g* misalignments in Section 3 and Section 4 (see Table 1 and Table 3). Results expressed in degrees.

Misalignment Angle	mg	vg	mv
ϕ	−0.192	0.117	≈−0.309
θ	0.009	−1.171	≈1.180
ψ	0.396	0.292	≈0.104

**Table 6:** Estimation results of (A) for three different figure eight manoeuvres driven on different days. M1 is the manoeuvre displayed in Figure 13 with $|{\tilde{f}}_{iv,y\;\max}^{m}|\approx 4$$\frac{m}{s^{2}}$, M2 represents a less dynamic Figure 8 drive with $|{\tilde{f}}_{iv,y\;\max}^{m}|\approx 3$$\frac{m}{s^{2}}$ and M3 corresponds with a more dynamic Figure 8 drive with $|{\tilde{f}}_{iv,y\;\max}^{m}|\approx 5$$\frac{m}{s^{2}}$.

Misalignment Angle	M1	M2	M3
$\phi_{\;mv}$	−0.313	−0.278	−0.300
$\theta_{\;mv}$	1.236	1.184	1.183
$\psi_{\;mv}$	0.037	0.058	0.045

## Appendix A. Derivation of (35)

Using the pitch and roll angles ($\theta_{\;rv}$, $\phi_{\;rv}$) with respect to the road plane and bearing in mind that they remain small, the vehicle velocities may be obtained from the over-road velocities as follows [3]:

(A1) $$\begin{array}{c} \begin{array}{cc} v_{ev,x}^{v} & =v_{\;er,x}^{r}-v_{\;rv,z}^{r}\;\theta_{\;rv}+h_{p}\;{\dot{\theta }}_{\;rv}\\ v_{ev,y}^{v} & =v_{\;er,y}^{r}+v_{\;rv,z}^{r}\;\phi_{\;rv}-h_{r}\;{\dot{\phi }}_{\;rv}\\ v_{ev,z}^{v} & =v_{\;rv,z}^{r}+v_{\;er,x}^{r}\;\theta_{\;rv}-v_{\;er,y}^{r}\;\phi_{\;rv} \end{array} \end{array}$$

where $h_{r}$ and $h_{p}$ are the height of the origin of the vehicle coordinate system with respect to the roll and pitch axes, respectively.

Both $v_{\;er,x}^{r}$ and $v_{\;rv,z}^{r}$ are primarily obtained from the odometry and suspension signals, respectively. $v_{\;er,y}^{r}$ could be provided by a sensor measuring the over-road lateral velocity, such as an optic sensor. Nevertheless, in the current work, we assume that such sensor is not available. Instead, this information is obtained from a single-track model, which provides a simplified but plausible description of the vehicle lateral motion within the region of lateral accelerations up to 4 $m/s^{2}$ under dry road conditions [37]:

(A2) $$v_{\;er,y}^{r}=l_{r}\;\omega_{z}^{r}-SG\;v_{\;er,x}^{r}\;f_{\;ir,y}^{r},$$

where the relationship between $f_{\;ir,y}^{r}$ and $f_{iv}^{v}$ may be expressed as follows

(A3) $$f_{\;ir,y}^{r}=f_{iv,y}^{v}-f_{iv,z}^{v}\;\phi_{\;rv}+{\ddot{\phi }}_{\;rv}\;h_{r}$$

Bearing in mind the low angular motion of land vehicles, particularly in the pitch and roll direction:

(A4) $$\begin{array}{cc} \omega_{z}^{r} & \approx \omega_{z}^{m} \end{array}$$

(A5) $$\begin{array}{cc} {\ddot{\phi }}_{\;rv} & \approx 0 \end{array}$$

(A6) $$\begin{array}{cc} f_{\;ir,y}^{r} & \approx f_{iv,y}^{r}=f_{iv,y}^{v}-f_{iv,z}^{v}\;\phi_{\;rv} \end{array}$$

Due to the misalignment error, the specific forces and angular rates supplied by the IMU are not expressed in *v* but in *m*. Using the rotation matrix $R_{\;v}^{\;m}$, the specific force is transformed from the set of resolving axes of coordinate system *m* to the set of resolving axes of coordinate system *v*

(A7) $$\begin{array}{c} f_{iv,y}^{v}=f_{iv,y}^{m}-\psi_{mv}\;f_{iv,x}^{m}+\phi_{mv}\;f_{iv,z}^{m}\\ f_{iv,z}^{v}=f_{iv,z}^{m}-\phi_{mv}\;f_{iv,y}^{m}+\theta_{mv}\;f_{iv,x}^{m} \end{array}$$

Furthermore, substituting (A7) in (A4) results in

(A8) $$f_{iv,y}^{r}=\left(f_{iv,y}^{m}-\psi_{mv}\;f_{iv,x}^{m}+\phi_{mv}\;f_{iv,z}^{m}\right)-\left(f_{iv,z}^{m}-\phi_{mv}\;f_{iv,y}^{m}+\theta_{mv}\;f_{iv,x}^{m}\right)\;\phi_{\;rv},$$

Now, substituting (A8) in (A2) yields

(A9) $$v_{\;er,y}^{r}=l_{r}\;\omega_{z}^{m}-SG\;v_{\;er,x}^{r}\;\left(f_{iv,y}^{m}-\psi_{mv}\;f_{iv,x}^{m}+\phi_{mv}\;f_{iv,z}^{m}-f_{iv,z}^{m}\;\phi_{\;rv}+\phi_{mv}\;f_{iv,y}^{m}\;\phi_{\;rv}-\theta_{mv}\;f_{iv,x}^{m}\;\phi_{\;rv}\right),$$

and combining (A9) and (A1) gives

(A10) $$\begin{array}{cc} v_{ev,x}^{v} & =v_{\;er,x}^{r}-v_{\;rv,z}^{r}\;\theta_{\;rv}+h_{p}\;{\dot{\theta }}_{\;rv}\\ v_{ev,y}^{v} & =l_{r}\;\omega_{z}^{m}-SG\;v_{\;er,x}^{r}\;\left(f_{iv,y}^{m}-\psi_{mv}\;f_{iv,x}^{m}+\phi_{mv}\;f_{iv,z}^{m}-f_{iv,z}^{m}\;\phi_{\;rv}+\phi_{mv}\;f_{iv,y}^{m}\;\phi_{\;rv}-\theta_{mv}\;f_{iv,x}^{m}\;\phi_{\;rv}\right)+v_{\;rv,z}^{r}\;\phi_{\;rv}-h_{r}\;{\dot{\phi }}_{\;rv}\\ v_{ev,z}^{v} & =v_{\;rv,z}^{r}+v_{\;er,x}^{r}\;\theta_{\;rv}\\ \; & -\left(l_{r}\;\omega_{z}^{m}-SG\;v_{\;er,x}^{r}\;\left(f_{iv,y}^{m}-\psi_{mv}\;f_{iv,x}^{m}+\phi_{mv}\;f_{iv,z}^{m}-f_{iv,z}^{m}\;\phi_{\;rv}+\phi_{mv}\;f_{iv,y}^{m}\;\phi_{\;rv}-\theta_{mv}\;f_{iv,x}^{m}\;\phi_{\;rv}\right)\right)\;\phi_{\;rv}, \end{array}$$

On the other hand, using the rotation matrix $R_{\;v}^{\;m}$ and applying the small-angle approximation, the following relationship is obtained:

(A11) $$\begin{array}{cc} v_{ev,x}^{m} & =v_{ev,x}^{v}-\psi_{mv}\;v_{ev,y}^{v}+\theta_{mv}\;v_{ev,z}^{v}\\ v_{ev,y}^{m} & =v_{ev,y}^{v}+\psi_{mv}\;v_{ev,x}^{v}-\phi_{mv}\;v_{ev,z}^{v}\\ v_{ev,z}^{m} & =v_{ev,z}^{v}-\theta_{mv}\;v_{ev,x}^{v}+\phi_{mv}\;v_{ev,y}^{v}, \end{array}$$

Rearranging (A11) yields

(A12) $$\begin{array}{cc} v_{ev,x}^{v} & =v_{ev,x}^{m}+\psi_{mv}\;v_{ev,y}^{v}-\theta_{mv}\;v_{ev,z}^{v}\\ v_{ev,y}^{v} & =v_{ev,y}^{m}-\psi_{mv}\;v_{ev,x}^{v}+\phi_{mv}\;v_{ev,z}^{v}\\ v_{ev,z}^{v} & =v_{ev,z}^{m}+\theta_{mv}\;v_{ev,x}^{v}-\phi_{mv}\;v_{ev,y}^{v} \end{array}$$

Substituting (A10) in (A12) results in

(A13) $$\begin{array}{cc} v_{x}^{v^{\prime }}+h_{p}\;{\dot{\theta }}_{\;rv}\; & =v_{ev,x}^{m}+\psi_{mv}\;v_{y}^{v^{\prime }}-\theta_{mv}\;v_{z}^{v^{\prime }}\\ v_{y}^{v^{\prime }}-h_{r}\;{\dot{\phi }}_{\;rv}-SG\;v_{\;er,x}^{r}\;\left(-\psi_{mv}\;f_{iv,x}^{m}+\phi_{mv}\;f_{iv,z}^{m}+\phi_{mv}\;f_{iv,y}^{m}\;\phi_{\;rv}-\theta_{mv}\;f_{iv,x}^{m}\;\phi_{\;rv}\right)\; & =v_{ev,y}^{m}-\psi_{mv}\;v_{x}^{v^{\prime }}+\phi_{mv}\;v_{z}^{v^{\prime }}\\ v_{z}^{v^{\prime }}+SG\;v_{\;er,x}^{r}\;\left(-\psi_{mv}\;f_{iv,x}^{m}+\phi_{mv}\;f_{iv,z}^{m}+\phi_{mv}\;f_{iv,y}^{m}\;\phi_{\;rv}-\theta_{mv}\;f_{iv,x}^{m}\;\phi_{\;rv}\right)\;\phi_{\;rv}\; & =v_{ev,z}^{m}+\theta_{mv}\;v_{x}^{v^{\prime }}-\phi_{mv}\;v_{y}^{v^{\prime }}, \end{array}$$

where

(A14) $$\begin{array}{cc} v_{x}^{v^{\prime }} & =v_{\;er,x}^{r}-v_{\;rv,z}^{r}\;\theta_{\;rv} \end{array}$$

(A15) $$\begin{array}{cc} v_{y}^{v^{\prime }} & =l_{r}\;\omega_{z}^{m}-SG\;v_{\;er,x}^{r}\;\left(f_{iv,y}^{m}-f_{iv,z}^{m}\;\phi_{\;rv}\right)+v_{\;rv,z}^{r}\;\phi_{\;rv} \end{array}$$

(A16) $$\begin{array}{cc} v_{z}^{v^{\prime }} & =v_{\;rv,z}^{r}+v_{\;er,x}^{r}\;\theta_{\;rv}-\left(l_{r}\;\omega_{z}^{m}-SG\;v_{\;er,x}^{r}\;\left(f_{iv,y}^{m}-f_{iv,z}^{m}\;\phi_{\;rv}\right)\right)\;\phi_{\;rv}, \end{array}$$

Finally, after taking into account the accelerometer biases, neglecting second-order error terms and rearranging, the measurement equations of (35) are obtained.
